# Robust elimination of genome-damaged cells safeguards against brain somatic aneuploidy following *Knl1* deletion

**DOI:** 10.1038/s41467-019-10411-w

**Published:** 2019-06-13

**Authors:** Lei Shi, Adel Qalieh, Mandy M. Lam, Jason M. Keil, Kenneth Y. Kwan

**Affiliations:** 1 Molecular & Behavioral Neuroscience Institute (MBNI), University of Michigan, Ann Arbor, MI 48109 USA; 2 Department of Human Genetics, University of Michigan, Ann Arbor, MI 48109 USA; 3 Medical Scientist Training Program, University of Michigan, Ann Arbor, MI 48109 USA

**Keywords:** Neural progenitors, Neural stem cells

## Abstract

The brain is a genomic mosaic shaped by cellular responses to genome damage. Here, we manipulate somatic genome stability by conditional *Knl1* deletion from embryonic mouse brain. *KNL1* mutations cause microcephaly and KNL1 mediates the spindle assembly checkpoint, a safeguard against chromosome missegregation and aneuploidy. We find that following *Knl1* deletion, segregation errors in mitotic neural progenitor cells give rise to DNA damage on the missegregated chromosomes. This triggers rapid p53 activation and robust apoptotic and microglial phagocytic responses that extensively eliminate cells with somatic genome damage, thus causing microcephaly. By leaving only karyotypically normal progenitors to continue dividing, these mechanisms provide a second safeguard against brain somatic aneuploidy. Without *Knl1* or p53-dependent safeguards, genome-damaged cells are not cleared, alleviating microcephaly, but paradoxically leading to total pre-weaning lethality. Thus, mitotic genome damage activates robust responses to eliminate somatic mutant cells, which if left unpurged, can impact brain and organismal fitness.

## Abstract (web summary)

The brain is a genomic mosaic shaped by somatic mutations and cellular responses that correct or purge these mutations. Here, by manipulating genome stability during embryonic brain development, authors revealed robust mechanisms that eliminate cells with genome damage from the brain, and the consequences of leaving somatic mutants unpurged

## Introduction

Maintenance of genome stability across divisions is essential to proliferating cells. In embryonic cerebral cortex, neurons and glia are generated by neural progenitor cells (NPCs) via successive rounds of cell divisions^1,2^. Somatic mutations inevitably arise during DNA synthesis and chromosome segregation, and their impact is alleviated by DNA repair, cell cycle checkpoints, and elimination of cells with genome damage^3,4^. Left uncorrected or unpurged, these mutations lead to somatic mosaicism^5,6^. The adult human brain harbors multiple types of somatic mosaic mutations, including aneuploidy^7–10^. Emerging evidence suggests that somatic mutations can contribute to brain disorders^11,12^. However, the mechanisms that curtail the propagation of brain somatic mutations, and the consequence of their impairment, remain incompletely understood.

Here, we generated a conditional allele of *Knl1* to manipulate genome stability in embryonic mouse brain, and leveraged this model to study the consequences of somatic genome instability in vivo. KNL1 (formerly CASC5 or BLINKIN) is a kinetochore component required for the spindle assembly checkpoint (SAC), which safeguards correct chromosomal segregation during mitosis^13,14^. In the presence of chromosomes unattached to microtubules, KNL1 functions as a scaffold for the assembly of the mitotic checkpoint complex, a potent inhibitor of anaphase. Upon secure attachment of all chromosomes, SAC is deactivated, and anaphase proceeds. In the absence of the KNL1-BUB3-BUB1 pathway, the ROD-ZWILCH-ZW10 pathway can activate SAC in response to unattached kinetochores, but SAC is deactivated prematurely, resulting in segregation errors^15^. Disruption of KNL1-BUB3-BUB1 components thus leads to numerical (whole chromosome) aneuploidy^7,14,16,17^.

Human mutations in *KNL1* are associated with autosomal recessive primary microcephaly (OMIM 604321)^18–21^. Previous studies of microcephaly have converged on mechanisms involving centrosome dysfunction^22^. *KNL1* is part of the emerging genetic data implicating altered SAC function in microcephaly^19,23^. However, the predicted outcome of SAC disruption, aneuploidy, is not particularly lethal to neural cells and has been reported to be prevalent in normal brain^24,25^. Aneuploidy, therefore, may not be the singular underlying cause of severe microcephaly in patients with *KNL1* mutations.

We find in mice that conditional deletion of *Knl1* from mitotic cortical NPCs leads to frequent chromosome segregation errors. The resulting missegregated chromosomes carry DNA damage in the form of double strand breaks (DSBs). Likely independently of aneuploidy, this DNA damage triggers rapid p53-dependent apoptotic and downstream microglial phagocytic responses that extensively eliminate the cells with chromosome missegregation. By leaving only karyotypically normal NPCs to continue dividing, these p53-dependent mechanisms provide a second safeguard against brain somatic aneuploidy in addition to the SAC. Applied en masse following *Knl1* deletion, however, they cause massive cell loss and severe microcephaly. In the absence of both *Knl1* and p53 safeguards against aneuploidy, genome-damaged cells are not eliminated from the brain. Paradoxically, this accumulation of somatic mutant cells, despite partially alleviating microcephaly, results in complete pre-weaning lethality. Our work thus unravels robust cellular processes in embryonic brain for eliminating cells with severe genome damage, which if left unpurged, can affect brain function and organismal fitness.

## Results

### *Knl1* deletion led to cortical NPC loss and microcephaly

Consistent with KNL1 function in the SAC^13,14^, publicly available single-cell RNA-seq^26,27^, and in situ hybridization^28^ data from embryonic cortex showed *Knl1* expression in proliferating NPCs in the germinal zones, the ventricular zone (VZ) and subventricular zone (SVZ), but not in post-mitotic neurons, and bulk RNA-seq^29,30^ showed forebrain *Knl1* expression throughout neurogenesis (Supplementary Fig. 1a). Constitutive deletion of *Knl1* in mice led to lethality on embryonic day (E)6 (MGI Ref. ID: J:175597). We recovered a conditional-ready lacZ reporter *Knl1* allele (*Knl1*^*tm1a*(EUCOMM)Hmgu^; Supplementary Fig. 1b) from frozen spermatozoa. LacZ reporter expression was confirmed in E15.5 cortex (Supplementary Fig. 1c), and *Knl1* conditional allele (*Knl1*^*fl/fl*^) was generated by crossing with Flpo recombinase mice to remove the lacZ cassette.

**Fig. 1:**
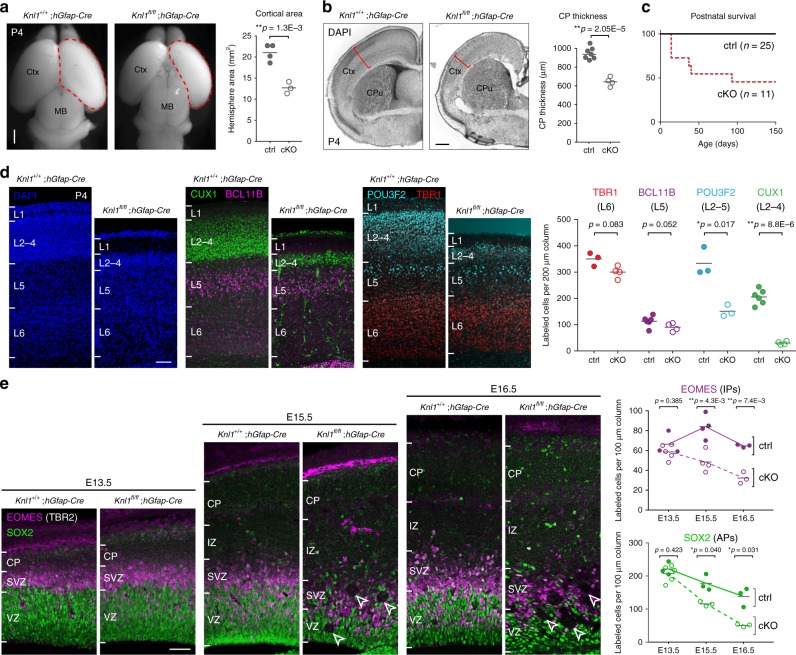
*Knl1* conditional deletion from cortical NPCs led to microcephaly and NPC loss. **a** Dorsal view of *Knl1*^*+l+*^;*hGFAP-Cre* and *Knl1*^*fl/fl*^;*hGFAP-Cre* (cKO) P4 brain. Cortical area was significantly reduced in cKO compared to littermate control (ctrl) (mean, two-tailed unpaired *t-*test, ctrl: *n* = 4, cKO: *n* = 3 animals, scale bar: 1 mm). **b** DAPI staining in coronal section of *Knl1*^*+l+*^;*hGFAP-Cre* and *Knl1*^*fl/fl*^;*hGFAP-Cre* (cKO) P4 brain. Cortical plate (CP) thickness was significantly reduced in cKO compared to ctrl (mean, two-tailed unpaired *t-*test, ctrl: *n* = 7, cKO: *n* = 4 animals, scale bar: 500 µm). **c** Postnatal survival analysis showed reduced survival in cKO (*n* = 11) compared to ctrl (*n* = 25 animals). **d** Cortical layer marker analysis at P4 revealed no significant change in BCL11B+ (L5, magenta) or TBR1+ (L6, red) neurons but a significant reduction in number of CUX1+ (L2–4, green) and POU3F2+ (L2–5, cyan) neurons in cKO compared to ctrl (mean, two-tailed unpaired *t-*test, TBR1, ctrl: *n* = 3, cKO: *n* = 4, BCL11B, ctrl: *n* = 6, cKO: *n* = 4, POU3F2, ctrl: *n* = 3, cKO: *n* = 3, CUX1, ctrl: *n* = 6, cKO: *n* = 4 animals, scale bar: 100 µm). **e** NPC marker analysis of E13.5 cortex revealed no significant change in cKO. At E15.5 and E16.5, SOX2+ apical progenitors (APs, green) and EOMES+ intermediate progenitors (IPs, magenta) were significantly reduced in number in cKO (mean, two-tailed unpaired *t-*test, EOMES E13.5, ctrl: *n* = 3 cKO: *n* = 5, E15.5, ctrl: *n* = 4, cKO: *n* = 4, E16.5, ctrl: *n* = 3, cKO: *n* = 3, SOX2 E13.5, ctrl: *n* = 3, cKO: *n* = 5, E15.5, ctrl: *n* = 3, cKO: *n* = 3, E16.5, ctrl: *n* = 3, cKO: *n* = 3 animals, scale bar: 50 µm). Aberrant gaps in immunolabeling (arrowheads) were observed in cKO. MB midbrain, CPu caudate putamen, IZ intermediate zone

To conditionally delete *Knl1*, we used *hGFAP-Cre*, a transgenic line that mediates Cre recombination in cortical NPCs starting at E12.5^31^. *Knl1*^*fl/fl*^;*hGFAP-Cre* (cKO) mice were viable at birth. On postnatal day (P)4 (Fig. 1a, b), compared to control (ctrl), cKO mice exhibited a 40% decrease in cortical area (ctrl, 21 ± 1.0 mm^2^; cKO, 13 ± 0.8mm^2^, mean ± s.e.m, *p* = 1.3E−3, two-tailed unpaired *t*-test) and a 32% decrease in cortical plate (CP) thickness (ctrl, 947 ± 9.1 µm; cKO, 643 ± 12.1 µm, *p* = 2.05E−5), consistent with microcephaly in patients with *KNL1* mutations. *Knl1* cKO mice were smaller in size and exhibited subviability, with ~75% survival at weaning (P21) and ~50% survival at P150 (*n* = 11 animals; Fig. 1c).

To investigate neuronal subtypes in cKO brain, we analyzed cortical layer markers^1^. At P4, TBR1-expressing layer (L)6 and BCL11B(CTIP2)-expressing L5 neurons were not significantly altered in number or laminar location (Fig. 1d). However, POU3F2-expressing L2–5 neurons were reduced by 55% (ctrl, 334 ± 31.2; cKO, 151 ± 12.8 per 200 µm column, *p* = 0.017, two-tailed unpaired *t*-test) and CUX1-expressing L2–4 neurons were reduced by 85% (ctrl, 206 ± 11.5; cKO, 30 ± 3.2 per 200 µm column, *p* = 8.8E−6). Agenesis of the corpus callosum was observed in cKO (Supplementary Fig. 1d). Thus, layer cell fates were specified following *Knl1* deletion, but upper layer (L2–4) neurons were selectively reduced, whereas deep layer (L5–6) neurons were not significantly affected.

In cortical neurogenesis, NPCs give rise first to deep layer neurons, then upper layer neurons^1^. The loss of late-born upper layer neurons is consistent with a progressive decrease in NPCs. We therefore analyzed NPCs at E13.5, E15.5, and E16.5. SOX2-expressing apical progenitors (APs) and EOMES(TBR2)-expressing intermediate progenitors (IPs) showed stage-dependent reductions in number, each exhibiting a significant loss by E15.5 (Fig. 1e) and a >50% reduction by E16.5 (EOMES: ctrl, 65 ± 0.9; cKO, 32 ± 3.3 per 200 µm column, *p* = 7.4E−3, SOX2, ctrl, 138 ± 16.6; cKO, 51 ± 2.1, *p* = 0.031, two-tailed unpaired *t*-test). Thus, deletion of *Knl1* led to progressive loss of NPCs. In E16.5 cKO cortex, some SOX2+ NPCs were positioned outside VZ and SVZ, suggesting a disorganization of embryonic cortical layering.

In addition to *hGFAP-Cre*, we conditionally deleted *Knl1* using *Emx1-Cre*^32^, which mediates Cre recombination in cortical NPCs starting at E10.5. Earlier *Knl1* deletion by *Emx1-Cre* led to microcephaly more severe than *hGFAP-Cre* (Supplementary Fig. 1e) and a loss of both deep and upper layer neurons (Supplementary Fig. 1f). This increased phenotypic severity is consistent with a progressive loss of NPCs following *Knl1* deletion and a requirement for *Knl1* by NPCs during both early and late neurogenesis. Together, these results suggested that *Knl1* deletion impaired NPC proliferation, survival, or maintenance.

### *Knl1* deletion led to rapid and robust apoptosis

**Fig. 2:**
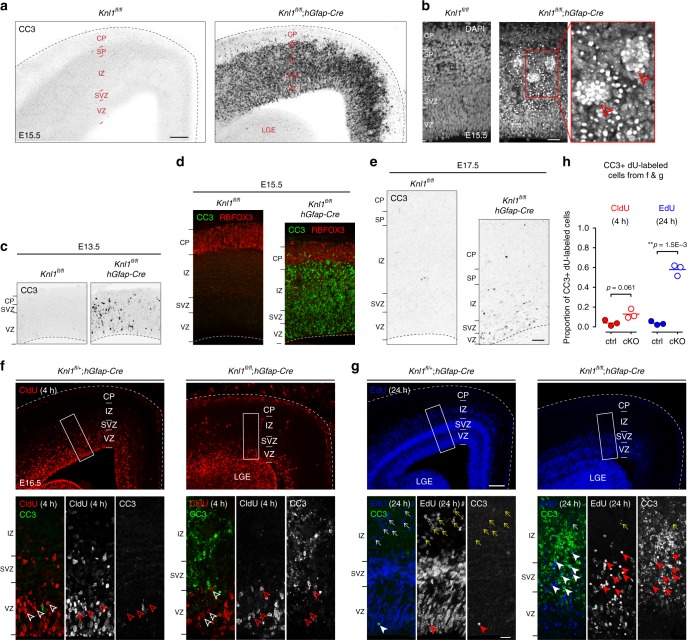
Robust and rapid apoptosis following *Knl1* deletion. **a** Cleaved Caspase 3 (CC3) immunostaining showed extensive apoptosis in E15.5 cKO cortex (scale bar: 100 µm). **b** DAPI staining revealed an abundance of pyknotic nuclei in E15.5 cKO cortex (scale bar: 50 µm). Many pyknotic nuclei exhibited a stereotyped clustering (arrowheads). **c** CC3 immunostaining showed that apoptosis was present in E13.5 cKO cortex, one day after the onset of *hGFAP-*Cre-mediated *Knl1* deletion at E12.5. **d** At E15.5, near the peak of neurogenesis, CC3 staining (green) was extensive in cortical VZ, SVZ, and intermediate zone (IZ), but largely absent from RBFOX3+ (NeuN+) cortical plate (CP) neurons (red) in cKO. **e** By E17.5, CC3 staining showed only a moderate increase in cKO compared to ctrl (scale bar: 50 µm). **f**, **g** Analysis of E16.5 cortex following a 4-h pulse of CldU (red) and a 24-h pulse of EdU (blue, scale bar: 100 µm). CC3 staining (green) showed no apoptosis in CldU+ cells (open arrowheads) in ctrl or cKO, but revealed extensive apoptosis in EdU+ cells (solid arrowheads) in cKO. EdU+ migrating neurons (yellow arrows) were present in the IZ of ctrl but largely absent from cKO (inset scale bar: 20 µm). **h** Quantification of CC3 colocalization with CldU or EdU (mean, two-tailed unpaired *t-*test, *n* = 3 animals). LGE lateral ganglionic eminence, h hours

To determine whether apoptosis contributed to NPC loss, we analyzed cleaved caspase 3 (CC3). Widespread CC3 labeling was observed in E15.5 cKO cortex, whereas only rare instances were found in littermate controls (Fig. 2a and Supplementary Fig. 2a). Consistently, DAPI DNA staining revealed an abundance of pyknotic nuclei, a condensation of chromatin and hallmark of apoptosis rarely found in controls (Fig. 2b and Supplementary Fig. 2b). In cKO, pyknotic nuclei showed a stereotyped clustering (arrowheads; Fig. 2b).

We next analyzed the progression of apoptosis in cKO. At E13.5, within one day of *hGFAP-Cre* onset^31^, CC3 was already increased in cortical wall, which at this age was mostly composed of NPCs, suggesting that apoptosis rapidly followed *Knl1* deletion from NPCs (Fig. 2c). At E15.5, near the peak of neurogenesis, CC3 was abundantly present in the germinal zones and intermediate zone (IZ), but was largely absent from CP, where RBFOX3(NeuN)-positive neurons had settled after migration (Fig. 2d). By E17.5, near the end of neurogenesis, CC3 staining was only marginally increased (Fig. 2e).

To assess the timing of apoptosis, we tracked NPC using a dual pulse of thymidine analogs 5-ethynyl-2′-deoxyuridine (EdU) and 5-chloro-2′-deoxyuridine (CldU) (Fig. 2f, g), which are incorporated into DNA in S-phase. CldU was given at E16.5, 4 h prior to analysis to label cells that recently entered S-phase. In cKO, CldU-labeled NPCs entered S-phase in VZ and SVZ, similar to littermate controls (open arrowheads, Fig. 2f). CldU+ nuclei in cKO were morphologically healthy and did not show significantly increased colocalization with CC3 compared to ctrl (Fig. 2h). Thus, following *Knl1* deletion, NPCs were able to enter S-phase and did not immediately undergo apoptosis. EdU was given at E15.5, to track cells that entered S-phase 24 h prior to analysis. In contrast to CldU, a majority of EdU+ cells had become apoptotic within 24 h of S-phase in cKO (solid arrowheads, Fig. 2g), with 58 ± 3.2% of EdU+ cells showing colocalization with CC3 compared to 4 ± 1.1% in control (Fig. 2h). To assess cell cycle progression, we used phospho-histone H3 (pHH3) staining to label M-phase cells and a 4-h pulse of CldU to label S-phase cells. This showed that per S-phase cell, the number of cells in M-phase was significantly increased by 2.7-fold in cKO (M-phase/S-phase ratio: ctrl, 7 ± 0.6%; cKO, 19 ± 0.3%, *p* = 0.036, two-tailed unpaired *t*-test, Supplementary Fig. 2c). The increase in pHH3+ cells was present by E13.5 (Supplementary Fig. 2d). This suggested that in cKO, a significantly increased proportion of cells was unable to progress normally through M-phase, which is in agreement with a recent in vitro study of a patient *KNL1* mutation^33^. Consistent with incompletion or delayed completion of mitosis, cKO cells largely failed to become post-mitotic neurons 24 h after S-phase. In control, many cells labeled with a 24 pulse of EdU were present in the IZ (yellow arrows, Fig. 2g), a region containing newly post-mitotic neurons migrating to the CP. In cKO, however, EdU-labeled cells largely failed to enter the IZ (Fig. 2g and Supplementary Fig. 2e). Together, these data revealed that cKO NPCs largely failed to complete mitosis or become migrating neurons within 24 h of S-phase, and a majority underwent apoptosis.

### *Knl1* deletion led to DNA damage and p53 activation in NPCs

**Fig. 3:**
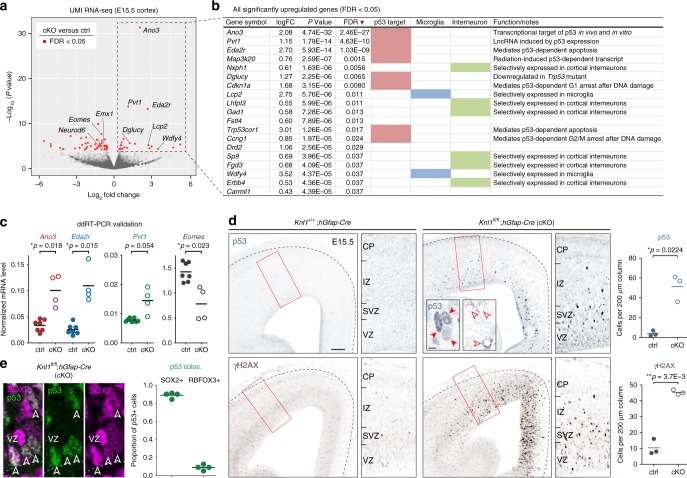
DNA damage and activation of p53 and p53 target genes in *Knl1* cKO brain. **a** Unique molecular identifier (UMI) RNA-seq volcano plot comparing cKO (*n* = 4) to ctrl (*n* = 7 animals) E15.5 cortex. *P*-value was calculated with likelihood ratio tests and false discovery rate (FDR) was calculated using the Benjamini–Hochberg procedure. Differentially expressed genes (FDR < 0.05) are indicated in red. **b** All 19 significantly upregulated genes. p53 target genes (pink), microglial genes (blue), and interneuronal genes (green) are indicated. **c** Droplet digital RT-PCR (ddRT-PCR) validated upregulation of p53 target genes *Ano3*, *Eda2r*, and *Pvt1*, and downregulation of *Eomes* in cKO compared to ctrl (mean, two-tailed unpaired *t-*test, ctrl: *n* = 7, cKO: *n* = 4 animals). **d** Immunostaining of E15.5 cortex revealed significant increases in the number of cells labeled by p53 or DNA damage marker γH2AX in cKO compared to ctrl (mean, two-tailed unpaired *t-*test, *n* = 3 animals, scale bar: 100 µm). p53 was localized to nuclei (solid arrowheads) and present in pyknotic cells (open arrowheads, inset scale bar: 5 µm). **e** Double immunostaining revealed p53 activation in SOX2+ NPCs in E15.5 cKO VZ (arrowheads). The vast majority of cells (89 ± 1.5%) with p53 activation were SOX2+ NPCs (mean, cKO: *n* = 4 animals). coloc. colocalization

Apoptosis is controlled by multiple regulatory mechanisms, including transcriptional regulation^34^. To gain insights into potential transcriptional mechanisms underpinning apoptosis in *Knl1* cKO, we performed transcriptome analysis. We reasoned that although apoptosis activates mRNA decay^35^, the initial transcriptional events that trigger apoptosis, and potential population changes in the transcriptome, can be detectable. We generated unique molecular identifier (UMI) RNA-seq libraries by Click-seq^36^ of cKO and littermate control cortices at E15.5, the peak of apoptosis in cKO. In Click-seq, UMI tagging of individual cDNA molecules enabled de-duplication of PCR-amplified reads. Analysis of ERCC spike-in RNA standards revealed excellent quantification within a dynamic range that covered essentially all analyzed genes (Supplementary Fig. 3a). Analysis of differential gene expression using edgeR^37^ revealed a total of 47 differentially expressed genes (68 transcripts including gene models) in cKO with a false discovery rate (FDR) of <0.05, (Fig. 3a, b and Supplementary Table 1). Strikingly, of the 19 upregulated genes, 8 are known to be transcriptionally activated by the tumor suppressor p53, bound at their genomic locus by p53, or both. Among the top three upregulated genes, *Ano3* is a target of p53 after X-irradiation^38^, *Eda2r* is activated in p53-dependent cell death^39^, and *Pvt1* is induced by p53^40^. Droplet digital RT-PCR (ddRT-PCR) validated upregulation of these genes (Fig. 3c), and showed an increase in *Bbc3* (*Puma*), a well-known p53-dependent mediator of apoptosis (Supplementary Fig. 3b). Additional genes at FDR < 0.1 included *Cdkn1a* (also known as p21) and *Ccng1*, which mediate p53-dependent cell cycle arrest. Our analysis also revealed changes in cell type composition. Among downregulated genes were those expressed by cells derived from the *hGFAP-Cre* lineage (e.g. *Eomes*, *Emx1*, Supplementary Table 1), which is consistent with apoptosis of cortical NPCs. Reduction in *Eomes* was confirmed by ddRT-PCR (Fig. 3c) and consistent with loss of IPs (Fig. 1e). Among upregulated genes were those specific to cells derived from interneuronal lineages unaffected by *hGFAP-Cre* (e.g. *Gad1*, *Erbb4*, Fig. 3b), a reflection of their relative increase following loss of *hGFAP-Cre*-derived NPCs. Together, these data reflected cell type-dependent apoptosis in *Knl1* cKO and supported p53-activated transcription as a pathway towards apoptosis.

In the absence of DNA damage, p53 is targeted by ubiquitin ligase MDM2 for degradation^41^. Following DNA damage, MDM2 is phosphorylated by ATM, which abrogates MDM2 degradation of p53^41^. We immunostained for stabilized p53, and found a 12-fold increase in the number of p53+ cells in cKO (Fig. 3d, ctrl, 4 ± 1.5; cKO, 51 ± 7.8 per 200 µm column, *p* = 0.0224, two-tailed unpaired *t*-test). p53 was localized to nuclei and present in pyknotic cells (Fig. 3d). To assess DNA damage, we immunostained for the histone variant γH2AX. The number of γH2AX+ cells was increased by 4.5-fold in cKO (Fig. 3d, ctrl, 10 ± 2.8; cKO, 45 ± 0.9 per 200 µm column, *p* = 0.0037). Furthermore, cKO brain was characterized by an increase in VZ cells labeled by phospho-(p)KAP1 (TRIM28) (Supplementary Fig. 3c), an ATM substrate and marker of heterochromatic DSBs. Notably, p53+, γH2AX+, or pKAP1+ cells were preferentially located in VZ and SVZ, and largely absent from CP, suggesting that DNA damage and p53 response occurred in NPCs. To directly identify cells with activated p53, we co-stained p53 with SOX2 (APs), EOMES (IPs), or RBFOX3 (neurons) in E15.5 cKO (Supplementary Fig. 3d). The vast majority of p53+ cells were SOX2+ (89 ± 1.5%, Fig. 3e), suggesting that DNA damage and p53 activation occurred in NPCs following *Knl1* deletion. Supporting this, γH2AX and p53 were widespread in cKO VZ by E13.5 (Supplementary Fig. 3e), when the majority of cortical cells were NPCs and preceding massive apoptosis at E15.5. Together, these analyses suggested that DNA damage, acquired in mitotic NPCs, elicited p53-dependent response following *Knl1* deletion.

### Missegregated chromosomes with DNA damage in *Knl1* cKO NPCs

**Fig. 4:**
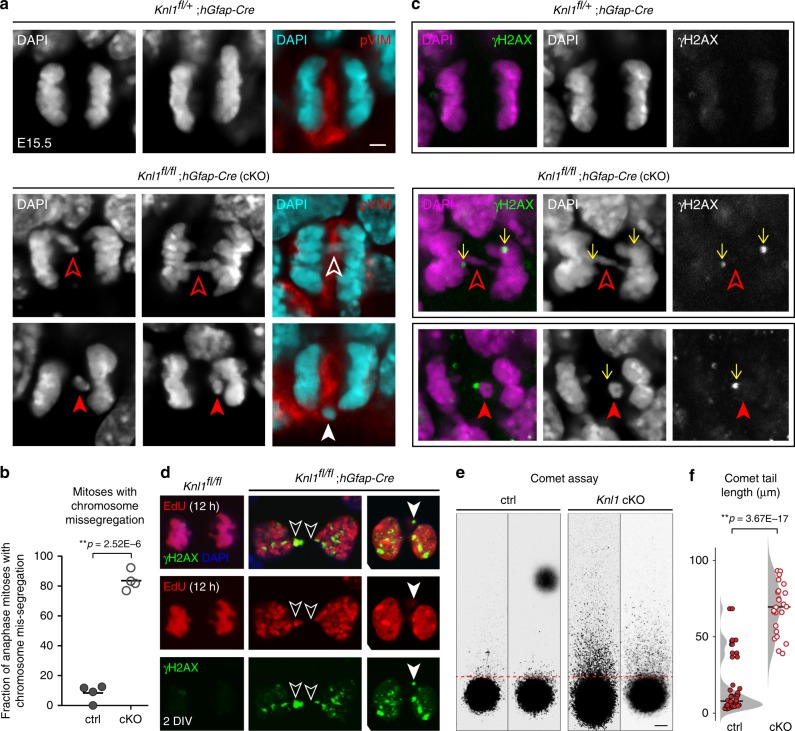
Missegregated chromosomes with DNA damage in mitotic NPCs following *Knl1* deletion. **a** DAPI staining revealed frequent lagging and bridged chromosomes (open arrowheads), and micronuclei (solid arrowheads) in anaphase NPCs in E15.5 cKO cortex. Missegregated chromosomes and micronuclei were present in the midzone labeled by phospho-Vimentin (pVIM, red, scale bar: 2 µm). **b** The percentage of anaphase cells with chromosome missegregation was significantly increased in cKO compared to ctrl (mean, two-tailed unpaired *t-*test, *n* = 4 animals). **c** Missegregated chromosomes and micronuclei in cKO were immunopositive for DNA damage marker γH2AX (green). **d** Dissociated E15.5 cortical NPC culture after 2 days in vitro (DIV) and a 12-h pulse of EdU (red). NPCs cultured from cKO were characterized by DNA bridges (open arrowheads) and micronuclei (solid arrowhead) immunopositive for γH2AX (green). **e**, **f** Single cell electrophoresis comet assay of E14.5 cKO and ctrl cortices (scale bar: 10 µm). Comet tail lengths were significantly increased in cKO compared to ctrl (mean, two-tailed unpaired *t-*test, ctrl: *n* = 36, cKO: *n* = 26 cells)

Disruption of SAC components causes chromosome missegregation and numerical aneuploidy^14,16,17^. Aneuploidy, however, can be tolerated by neural cells^24,25^ and does not inevitably lead to p53 activation^42^. Interestingly, errors in segregation can give rise to lagging chromosomes at the cleavage furrow that are prone to DNA damage^42–44^. We reasoned that missegregation-related chromosome damage may be present in *Knl1* cKO. We therefore surveyed mitotic figures at the ventricular surface, where apical NPCs undergo mitosis (Fig. 4a and Supplementary Fig. 4a). In control E15.5 VZ, we found an abundance of anaphase cells, only a minority of which exhibited potential chromosome missegregation. In striking contrast, in cKO, 83.6% of anaphase cells were characterized by lagging or bridged chromosomes (open arrowheads, Fig. 4a, b, ctrl, 8.3 ± 2.87%; cKO, 83.6 ± 3.21%) within the phospho-Vimentin(pVim)-labeled spindle midzone (Fig. 4a and Supplementary Fig. 4b). In addition, micronuclei, which are chromosomes not incorporated into either daughter nuclei and a hallmark of chromosomal instability^45^, were present in mitotic NPCs (solid arrowheads, Fig. 4a). Notably, γH2AX+ foci were consistently found within or near lagging chromosomes and in micronuclei (arrows, Fig. 4c and Supplementary Fig. 4c), and the proportion of metaphases with γH2AX foci was significantly increased in cKO (ctrl, 14 ± 1.2%; cKO, 74 ± 4.4%, Supplementary Fig. 4d). To better visualize chromosomal defects, we used NPC cultures from E15.5 cortices. One day after dissociation, we applied EdU for 12 h to label mitotic NPCs prior to fixation. Using EdU to visualize DNA, we observed an abundance of DNA bridges with γH2AX (Fig. 4d and Supplementary Fig. 4e), which were often localized near the cleavage furrow, suggesting that DNA damage may be associated with cytokinesis^44^.

To directly assay for DNA breaks, we performed single cell electrophoresis comet assay on E14.5 cortices. This showed in cKO a significant increase in comet tailing (ctrl, 8.9 ± 1.96%; cKO, 34.1 ± 3.74%, *p* = 0.0175, two-tailed unpaired *t*-test, Fig. 4e and Supplementary Fig. 4f) and tail length among cells with tails (ctrl: 14.5 ± 2.67 µm, cKO: 68.6 ± 3.31 µm, *p* = 3.67E−17, Fig. 4f), indicating a considerable increase in DSBs. Together, chromosome missegregation, increased DNA breaks, and activation of p53 in NPCs strongly supported mitotic DSBs as an important trigger for p53-dependent response in *Knl1* cKO.

### *Trp53/Knl1* co-deletion partially rescued *Knl1* cKO phenotypes

**Fig. 5:**
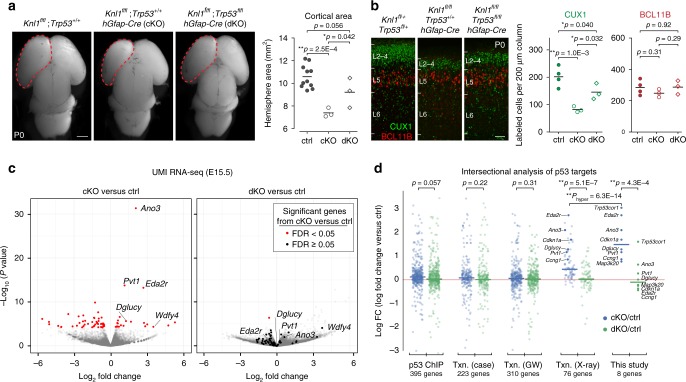
Co-deletion of *Trp53* with *Knl1* partially ameliorated *Knl1* phenotypes. **a** Dorsal view of *Knl1*^*fl/+*^*;Trp53*^*fl/+*^, *Knl1*^*fl/fl*^*;Trp53*^*+/+*^*;hGfap-Cre* (cKO), and *Knl1*^*fl/fl*^*;Trp53*^*fl/fl*^*;hGfap-Cre* (dKO) P0 brain (scale bar: 1 mm). The significant cortical area loss in cKO compared to littermate control (ctrl) was partially ameliorated in dKO (mean, ANOVA with post-hoc *t-*test, ctrl: *n* = 11, cKO: *n* = 3, dKO: *n* = 3 animals). **b** Cortical layer marker analysis at P0 revealed no significant change in BCL11B+ (L5, red) neurons (scale bar: 50 µm). The reduction in CUX1+ (L2–4, green) neuron number in cKO compared to ctrl was partially ameliorated in dKO (mean, ANOVA with post-hoc *t-*test, ctrl: *n* = 4, cKO: *n* = 3, dKO: *n* = 3 animals). **c** UMI RNA-seq volcano plot comparing cKO to ctrl, and dKO to ctrl E15.5 cortex (ctrl: *n* = 7, cKO: *n* = 4, dKO: *n* = 4 animals). In the cKO versus ctrl comparison, differentially expressed genes (FDR < 0.05) are indicated in red (left panel). The same genes were labeled in the dKO versus ctrl comparison (right panel). One gene (gene model Gm29260) showed differential expression in dKO (FDR < 0.05) and is indicated in red. The other genes did not show significant differential expression in dKO (FDR ≥ 0.05) and are indicated in black. **d** Intersectional analysis of p53 target genes identified by p53 ChIP, case studies of p53 transcriptional regulation (Txn. case), genome wide studies of p53 transcriptional regulation (Txn. GW), and ionizing radiation-induced p53 transcriptional regulation (Txn. X-ray). p53-dependent genes induced by ionizing radiation were upregulated in cKO versus ctrl, and showed a significant enrichment (mean, two-tailed paired *t-*test and hypergeometric test)

To understand the necessity of p53 in the cKO apoptotic response and the consequences of disrupting both SAC and p53-dependent mechanisms, we generated *Knl1/Trp53* conditional double mutants (*Knl1*^*fl/fl*^*;Trp53*^*fl/fl*^*;hGFAP-*Cre, dKO). Analysis of *Knl1/Trp53* dKO brain revealed partial amelioration of phenotypes found in *Knl1* single cKO. At P0 and P4, cortex size was partially restored (P0: ctrl, 11 ± 0.3; cKO, 7 ± 0.2; dKO, 9 ± 0.8 mm^2^, ctrl:cKO, *p* = 2.5E−4; ctrl:dKO, *p* = 0.056; cKO:dKO, *p* = 0.042, ANOVA with post-hoc *t*-test, Fig. 5a and Supplementary Fig. 5a). Loss of CUX1+ neurons also was partially ameliorated (ctrl, 202 ± 18.2; cKO 82 ± 6.2; dKO 146 ± 16.9 per 200 µm column, ctrl:cKO, *p* = 1.0E−3; ctrl:dKO, *p* = 0.040; cKO:dKO, *p* = 0.032, Fig. 5b and Supplementary Fig. 5b). NPCs number was not significantly decreased in dKO compared to control, which is consistent with partial alleviation of microcephaly (Supplementary Fig. 5c).

To analyze whether the p53-dependent transcriptome signature we found in cKO remained, we performed UMI RNA-seq of E15.5 dKO cortex (Fig. 5c and Supplementary Table 2). This revealed a striking reversal of essentially all gene expression changes observed in *Knl1* cKO (Supplementary Table 3). Among the 68 differentially regulated transcripts we found in cKO compared to control (FDR < 0.05), only one transcript (gene model Gm29260) remained significant in dKO compared to control. Importantly, the eight significantly upregulated p53 targets from cKO were each at an FDR of 1.000 (i.e. not differentially expressed) in dKO (Supplementary Fig. 5d). These results were validated using ddRT-PCR (Supplementary Fig. 5e). Overall, both upregulated and downregulated genes at FDR < 0.05 from cKO versus control became normalized following co-deletion of *Trp53*, shifting in log-fold change towards 0 (i.e. not differentially expressed) (Supplementary Fig. 5f). Therefore, the transcriptome changes in cKO were largely p53-dependent.

p53 regulates diverse target genes in a context-dependent manner^46,47^. Intersectional analyses (Fig. 5d) showed that, taken as a single group, neither genes bound by p53 nor p53 transcriptional targets^48^ were differentially regulated in cKO compared to dKO. However, intersectional analysis with genes activated by X-radiation-induced DSBs^38^ were significantly overrepresented in our top p53-dependent genes in cKO (*P*_hyper_ = 6.3E−14, hypergeometric test) and showed a significant difference between cKO and dKO (*p* = 5.1E−7, two-tailed paired *t*-test). Thus, not all p53-dependent genes were upregulated following *Knl1* deletion, and the transcriptomic signature of p53 activation in cKO is consistent with DSBs as a trigger.

### Attenuated apoptosis following *Knl1*/*Trp53* co-deletion

**Fig. 6:**
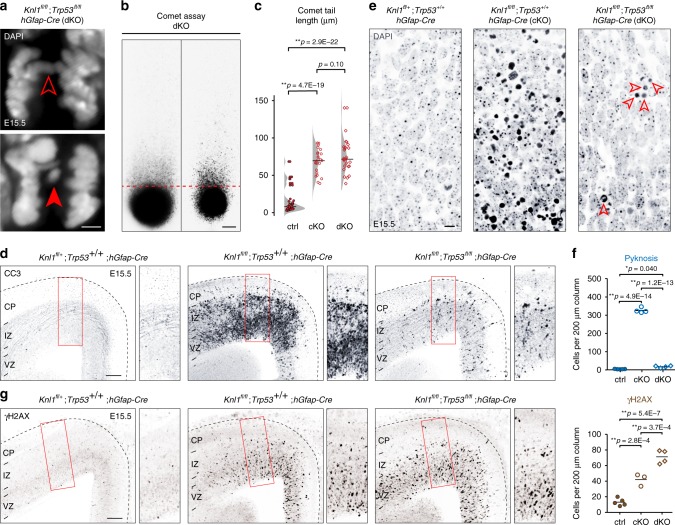
Persistent DNA damage and attenuated apoptosis in *Knl1*/*Trp53* dKO. **a** In E15.5 dKO brain, DAPI staining revealed lagging chromosomes (open arrowheads) and micronuclei (red solid arrowhead) in anaphase NPCs similar to those in cKO (scale bar: 2 µm). **b** Single cell electrophoresis comet assay of E14.5 dKO cortices (scale bar: 10 µm). **c** Comet tail lengths were significantly increased in dKO compared to ctrl (mean, ANOVA with post-hoc *t* test, ctrl: *n* = 36, cKO: *n* = 26, dKO: *n* = 24 cells). **d** CC3 immunostaining in E15.5 cortex showed reduced apoptosis in dKO compared to cKO (scale bar: 100 µm). **e**, **f** DAPI staining in E15.5 dKO cortex revealed pyknotic nuclei (open arrowheads), which was increased in number in dKO compared to ctrl (scale bar: 10 µm). This increase, however, was significantly smaller than the increase in cKO (mean, ANOVA with post-hoc *t-*test, ctrl: *n* = 5, cKO: *n* = 4, dKO: *n* = 4 animals). **g** Immunostaining of E15.5 cortex revealed a significant increase in the number of γH2AX+ cells in dKO compared to cKO or ctrl (mean, ANOVA with post-hoc *t-*test, ctrl: *n* = 5, cKO: *n* = 4, dKO: *n* = 4 animals), indicating that without p53, apoptosis in dKO was less efficient at clearing genome-damaged cells (scale bar: 100 µm)

Although co-deletion of *Trp53* abolished the p53-dependent transcriptional response, chromosome missegregation from SAC disruption should persist in dKO. Indeed, we found significant increases in chromosome missegregation and DNA breaks in dKO (Fig. 6a–c and Supplementary Fig. 6a). Next, we investigated whether apoptosis could be elicited in the absence of p53 (Supplementary Fig. 6c). We found an increase in CC3 immunostaining in E15.5 dKO compared to control (Fig. 6d), but that increase was modest compared to the p53-intact cKO. Pyknosis was also present (Fig. 6e, f, ctrl, 4 ± 0.6; cKO, 327 ± 6.9; dKO, 18 ± 2.7 per 200 µm column). But compared to cKO, pyknosis was not increased to the same extent (dKO 18-fold lower than cKO, *p* = 1.2E−13, ANOVA with post-hoc *t*-test), suggesting that although apoptosis was present, it was less efficient compared to p53-dependent mechanisms. This was supported by reduced clearance of γH2AX+ cells (Fig. 6g). Compared to cKO, γH2AX+ cells were 1.7-fold more numerous in dKO (cKO, 42 ± 2.6, dKO, 71 ± 4.3 per 200 µm column, *p* = 3.7E−4). Cells labeled by heterochromatic DSB marker pKAP1 were also less efficiently cleared (Supplementary Fig. 6d). Together, these results showed that cells with genome damage as a result of *Knl1* deletion underwent p53-dependent apoptosis in cKO. In the absence of p53 in dKO, apoptosis occurred at significantly lower levels and was less efficient at clearing cells with genome damage.

### Robust phagocytosis of apoptotic cells by microglia in *Knl1* cKO

**Fig. 7:**
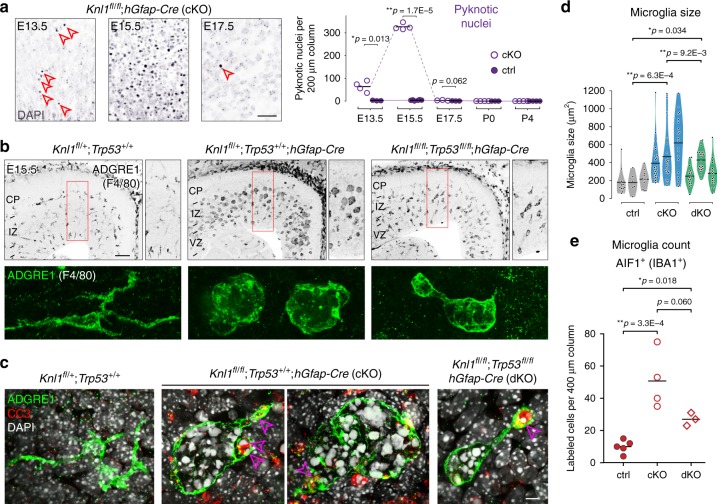
Phagocytosis of apoptotic cells by activated microglial in *Knl1* cKO and *Knl1*/*Trp53* dKO. **a** Developmental analysis by DAPI staining revealed that pyknotic nuclei (open arrowheads) were present at E13.5, peaked in number at E15.5, and eliminated by birth in cKO cortex (mean, two-tailed unpaired *t-*test, E13.5, ctrl: *n* = 4 cKO: *n* = 3, E15.5, ctrl: *n* = 4, cKO: *n* = 6, E17.5, *n* = 3, P0, ctrl: *n* = 4, cKO: *n* = 3, P4, *n* = 4 animals, scale bar: 50 µm). **b** ADGRE1 (F4/80) immunostaining revealed morphologic activation of microglia in E15.5 cKO and dKO cortex (scale bar: 100 µm). Confocal z-stacking revealed that ADGRE1+ (green) microglia acquired a ballooned morphology. **c** ADGRE1+ (green) microglia with ballooned morphology phagocytosed dozens of DAPI+ (white) pyknotic nuclei, some of which were CC3+ (red, and open arrowheads) in cKO and dKO cortex at E15.5 (scale bar: 5 µm). **d** Phagocytic microglia in cKO and dKO were significantly larger in size compared to ctrl microglia. However, the increase in microglial size was significantly attenuated in dKO compared to cKO (mean, ANOVA with post-hoc *t-*test, *n* = 3 animals). **e** The number of AIF1+ (IBA1+) microglia was significantly increased in cKO and dKO compared to ctrl. The increase in microglial number, however, was attenuated in dKO compared to cKO (mean, ANOVA with post-hoc *t* test, ctrl: *n* = 5, cKO: *n* = 4, dKO: *n* = 3 animals)

In *Knl1* cKO, we found an abundance of pyknotic nuclei compared to controls in the embryonic brain that were cleared by early postnatal ages (Fig. 7a), implicating a robust mechanism for their elimination. In vivo, post-apoptotic cells are eliminated via phagocytosis, by macrophages in peripheral tissues. Our RNA-seq of cKO showed significant increases in *Lcp2* and *Wdfy4*, two genes selectively expressed in microglia (Fig. 3b and Supplementary Table 1)^26,49^. Microglia are resident phagocytes present in embryonic brain as early as E10.5^50,51^. To analyze microglia, we immunostained for ADGRE1 (F4/80), a marker of macrophagic cells. At E15.5, we found a striking change in microglial morphology (Fig. 7b). Confocal z-stack analysis revealed that microglia acquired a ballooned morphology. Each ballooned microglia contained dozens of phagocytosed pyknotic nuclei (Fig. 7c), which explained their stereotyped clustering (Fig. 2b). Some of the pyknotic nuclei were CC3+, confirming that they were apoptotic cells (Fig. 7c). In cKO, microglial size was increased by 2.5-fold (Fig. 7d, ctrl, 189.8 ± 21.29; cKO, 481.1 ± 35.17 µm^2^, *p* = 6.3E−4, ANOVA with post-hoc *t*-test) and analysis using microglia marker AIF1 (IBA1) showed similar changes (Supplementary Fig. 7a). Microglial number was also increased, by approximately five-fold (Fig. 7e, ctrl, 10 ± 1.8; cKO, 51 ± 8.9 per 400 µm column, *p* = 3.3E−4). Microglia have self-renewal capacity^50,51^. At this embryonic age, microglia were proliferative and marked by KI67 in ctrl and cKO (Supplementary Fig. 7b), suggesting that their abundance could result from proliferation. By P0, concomitant with clearance of pyknotic nuclei (Fig. 7a), ADGRE1+ microglia in cKO became comparable to control in size and morphology, and were mononuclear (arrowheads, Supplementary Fig. 7c). Together, these results are consistent with robust microglial activation following massive apoptosis, leading to rapid elimination of post-apoptotic cells from the prenatal *Knl1* cKO brain.

To determine whether the microglial response was associated with p53, we examined E15.5 dKO. We found significant increases in both number and size of microglia compared to control (Fig. 7c–e). These increases, however, were attenuated compared to those in cKO (Fig. 7d, e; size: cKO, 481.1 ± 35.17 µm^2^; dKO, 325.4 ± 19.19 µm^2^, *p* = 9.2E−3; number: cKO, 51 ± 8.9; dKO, 27 ± 2.3 per 400 µm column, *p* = 0.060, ANOVA with post-hoc *t* test), and consistent with significantly lower levels of apoptosis in dKO (Fig. 6d–f). Thus, our analyses of *Knl1/Trp53* dKO showed that chromosomal damage can lead to apoptotic and microglial phagocytic responses via a p53-independent mechanism. However, in the absence of p53, these responses were attenuated and significantly less efficient at clearing genome-damaged cells, thus partially alleviating microcephaly associated with *Knl1* deletion.

### Unpurged somatic mutants and total pre-weaning lethality in dKO

**Fig. 8:**
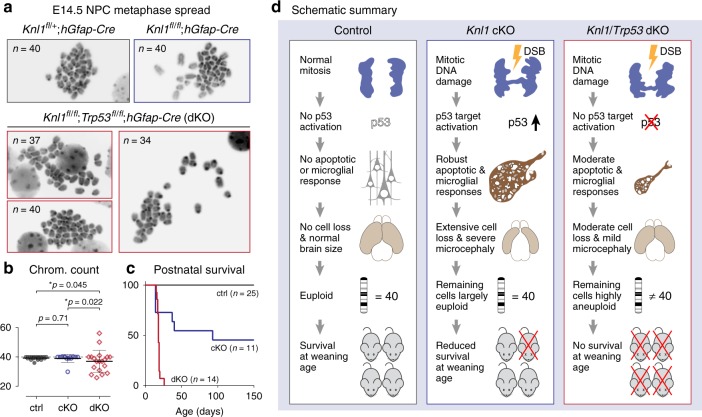
Persistent somatic mutant cells and complete pre-weaning lethality in *Knl1*/*Trp53* dKO. **a**, **b** Karyotype analysis of E14.5 NPCs by metaphase spread. Mitotic NPCs in cKO largely had normal karyotypes similar to ctrl. In contrast, 79% of mitotic NPCs in dKO were aneuploid (mean, error bars: ±s.e.m., Fisher’s exact test, ctrl: *n* = 19, cKO: *n* = 13, dKO: *n* = 19 metaphase spreads). **c** Survival analysis of ctrl, cKO, and dKO showed complete pre-weaning lethality in dKO (ctrl: *n* = 25, cKO: *n* = 11, dKO: *n* = 14 animals). **d** Schematic summary. In *Knl1* cKO, DNA damage on missegregated chromosomes in mitotic NPCs were a potent trigger for rapid p53-dependent apoptotic and downstream microglial mechanisms that extensively cleared the NPCs with segregation errors, leading to massive cell loss and microcephaly. This clearance left only karyotypically normal NPCs to continue dividing. In *Knl1*/*Trp53* dKO, DNA damage on missegregated chromosomes was unable to elicit robust apoptotic or microglial responses, thus leaving genome-damaged and aneuploid NPCs unpurged and able to continue dividing. This alleviated the severity of microcephaly, but paradoxically resulted in complete pre-weaning lethality. Chrom. chromosome

To assess the efficiency with which p53-dependent mechanisms cleared genome-damaged cells following *Knl1* deletion, we karyotyped NPCs from E14.5 cortex. Remarkably, mitotic NPCs in cKO had normal karyotypes comparable to control (chromosome number, *p* = 0.71, Fisher’s exact test, Fig. 8a, b), suggesting that cells with DNA damage from a previous round of mitosis were largely cleared by p53-dependent responses prior to the next cell cycle. The remaining mitotic cells, therefore, were karyotypically normal. Qualitatively, we observed that the number of metaphase spreads were lower in cKO compared to control, which was consistent with loss of NPCs in cKO. To determine the genomic consequences of *Knl1* deletion in the absence of p53, we karyotyped NPCs from dKO. Strikingly, aneuploidy was found in the majority of dKO NPCs (79%, Fig. 8a, b), suggesting that in the absence of p53, NPCs with missegregation were not efficiently cleared and continued dividing. Consistent with this, microcephaly as a result of *Knl1* deletion was partially alleviated in dKO (Fig. 5a). Paradoxically, despite partial restoration of brain size, survival analysis of dKO revealed complete pre-weaning lethality, compared to about 25% cKO lethality at the time of weaning (dKO survival 0/14; cKO survival 8/11, Fig. 8c).

Together, these results showed that, following *Knl1* deletion and SAC disruption, DNA damage on missegregated chromosomes triggered rapid p53-dependent apoptotic and downstream microglia phagocytic responses that efficiently eliminated cells with chromosome missegregation from embryonic brain, thus causing severe microcephaly (Fig. 8d). However, because genome-damaged cells were cleared, the remaining cells were largely euploid, and cKO organismal survival at weaning was at ~75%. Following co-deletion of *Trp53* with *Knl1*, genome-damaged cells were not efficiently cleared and somatic mutant cells remained in the brain. Despite partial alleviation of microcephaly, the dKO showed total pre-weaning lethality. This paradox suggested that severely genome-damaged cells, if present in substantial numbers in the brain, can impact organismal fitness.

## Discussion

Genomic mosaicism in the brain is the combined outcome of cumulative developmental and post-developmental somatic mutations, and cellular responses that correct or purge these mutations^3,5,6^. In this study, we manipulated somatic genome stability during embryonic brain development and gained insights into the robust mechanisms that eliminate cells with somatic genome damage from the brain, and the consequences of leaving somatic mutant cells unpurged.

Mammalian cells lack an aneuploidy checkpoint, and thus rely on the SAC to ensure correct chromosome segregation during mitosis as a safeguard against aneuploidy. By conditional deletion of *Knl1* from NPCs, we found that disruption of the SAC did not inevitably lead to brain somatic aneuploidy. In mitotic NPCs, DNA damage on missegregated chromosomes was a potent trigger for rapid p53-dependent apoptotic and microglial mechanisms that efficiently cleared cells with segregation errors, thus leaving only karyotypically normal NPCs to continue dividing. Protection against brain somatic aneuploidy is therefore multilayered: (1) SAC delays anaphase onset until secure microtubule attachment on all chromosomes and (2) in the event of SAC disruption in NPCs, DNA damage on the resulting missegregated chromosomes triggers p53-dependent mechanisms to remove these cells. In the normal brain, these mechanisms rapidly eliminate the small proportion of NPCs with such genome damage, thereby maintaining genome stability. Applied en masse, as in *Knl1* deletion, apoptotic elimination of these cells would lead to massive cell loss and microcephaly. In normal adult brain, the frequency of numerical aneuploidy remains controversial, with rates ranging from 0% to 47% in human brain and 0% to 57% in mouse brain^52^. Brain somatic aneuploidy arises from segregation errors in NPCs. Our finding that the resulting missegregated chromosomes carry DNA damage that triggers elimination of damaged cells is thus consistent with brain-derived somatic aneuploidy being rare in normal brain^14^.

KNL1 is expressed in mitotic cells and functions in the SAC, a mitotic checkpoint^13,14^. This role of KNL1 and the segregation errors in cKO NPCs convergently support mitosis as the point of primary deficit that ultimately elicited robust apoptotic responses following *Knl1* deletion. This is consistent with our result that a majority of cells became apoptotic within 24 h of S-phase (Fig. 2). Furthermore, a human *KNL1* mutation causes mitotic defects and cell death in hESC-derived neural progenitors^33^, providing support that the mitotic function of KNL1 in NPCs is conserved. We have not excluded the possibility that apoptosis can also occur post-mitosis after initial mitotic segregation errors. The minority of cells that were not rapidly eliminated within this time frame may undergo apoptosis by other mechanisms, including cell cycle delay. In addition, although microglia phagocytosis is efficient, following massive apoptosis in cKO, clearance of post-apoptotic cells may be delayed, thus explaining transient accumulation of cleaved caspase staining outside the germinal zones (Fig. 2).

Collectively, the DNA damage on missegregated chromosomes, the activation of p53 in NPCs, and the p53-DSB transcriptomic signature converge on DNA DSBs as the key trigger for rapid p53 activation following *Knl1* deletion. The precise mechanisms that generate DSBs following missegregation remain unknown and would require technical advances from the field to resolve. There are several possibilities. Lagging chromosomes trapped in the cleavage furrow can acquire DSBs during cytokinesis^44^. However, physical cutting by the furrow is unlikely, as chromosome bridges would trigger the abscission checkpoint^53^. Micronuclei, formed when lagging chromosomes are excluded from either daughter nuclei, can also carry DNA damage^43,54^. It has been proposed that the micronuclear envelope is liable to rupture, which can expose DNA to cytoplasmic nucleases^42^, thus contributing to chromosomal pulverization and chromothripsis^43,54^. Consistent with this, we found no evidence of persistent micronuclei in postnatal cKO brain, suggesting that they had been eliminated by apoptosis following p53 activation. Furthermore, following SAC disruption, anaphase can proceed prematurely in the presence of under-replicated or insufficiently decatenated DNA, ultimately leading to ultrafine bridges^55^ that can contribute to DNA damage if not resolved during mitosis.

Although aneuploidy has been associated with p53 upregulation, whether aneuploidy itself can be the trigger for rapid p53 activation is debated^14,56^. Two recent studies have shown that numerical aneuploidy does not inevitably cause p53 activation^42,57^. However, p53 does respond to structural aneuploidies generated by segregation errors and chromosomal breaks. Consistent with these emerging data, *Knl1* deletion in vivo led to DNA damage on missegregated chromosomes and rapid apoptosis, suggesting that mitotic DNA damage, not aneuploidy itself, triggered the rapid p53-dependent responses. Further supporting this, in *Bub1b*^*H/H*^ hypomorphic mutant mice, widespread aneuploidy present in ~40% of brain cells^7,58^ did not cause massive apoptosis or developmental microcephaly^59^. However, we have not excluded the possibility that in the longer term, aneuploidy can contribute to apoptosis.

To investigate the consequence of disrupting both the SAC and p53-dependent safeguards against brain somatic aneuploidy, we co-deleted *Trp53* with *Knl1*. Following *Trp53* co-deletion, segregation errors and DNA damage persisted, but the robust transcriptional, apoptotic, and microglial phagocytic responses were largely abrogated. Without p53, NPCs with chromosome missegregation were not eliminated and widespread mosaic aneuploidy was present, thus confirming p53-dependent mechanisms as a second safeguard against brain somatic aneuploidy. Without massive elimination of somatic mutant cells, *Trp53* co-deletion partially alleviated microcephaly resulting from *Knl1* deletion, but paradoxically led to complete pre-weaning lethality. The precise cause of dKO lethality, however, is unknown. At the cellular level, aneuploidy, unrepaired DNA damage, or both could contribute. At the organismal level, dKO pups were able to breathe and suckle, and we have not been able to ascertain potential causes of pre-weaning death. Our results, however, do strongly support the possibility that somatic mutant cells derived from divisions in the absence of both SAC and p53-dependent safeguards, if present in substantial numbers, can affect brain and organismal fitness.

Altered SAC function has only recently emerged as a mechanism of microcephaly^19,23^. We found that SAC disruption in *Knl1* cKO led to microcephaly, but likely not as a direct result of aneuploidy. Instead, SAC disruption caused frequent segregation errors in NPCs. DNA damage on the resulting missegregated chromosomes triggered robust removal of damaged cells, resulting in large-scale cell loss and microcephaly. Interestingly, centrosomal deficit can cause microcephaly and activate p53 without increasing γH2AX^60^, suggesting that centrosomal dysfunction does not inevitably cause DSBs, unlike *Knl1* deletion. These results thus highlight mechanistic differences between SAC disruption and centrosomal dysfunction in microcephaly.

Microglia have emerged as key players in CNS development, homeostasis, and disease^50,51,61^. In *Knl1* cKO, microglia underwent morphologic activation, increased in number, and exhibited a remarkable capacity for eliminating apoptotic cells with genome damage. Our work thus implicated a novel role for microglia in maintaining genome stability during embryonic development. Interestingly, recent work suggested that microglia can induce apoptosis of brain cells^51,61^. Thus, in vivo, microglia may accelerate apoptosis in addition to clearing apoptotic debris, playing more than one role in curtailing the propagation of cells with genome damage in the brain.

## Methods

### Mouse lines

A conditional-ready lacZ reporter *Knl1* allele (*Knl1*^*tm1a*(EUCOMM)Hmgu^; EUCOMM (HEPD0665_2_E05)) was obtained from EUCOMM and recovered from cryopreserved spermatozoa at the University of Michigan Transgenic Core. The *Knl1*^*fl/fl*^ conditional allele was generated by crossing with a Flpo recombinase transgene to remove the neomycin resistance gene cassette from the germline via FRT site recombination. Neomycin cassette-deletion was confirmed by PCR. The *Knl1*^fl/fl^ mice were used to generate conditional mutants and genotyped by PCR. The mice used in this study were: *p53*^*LoxP*^ (The Jackson Laboratory, Stock No. 008462), *ROSA26*^*Flpo*^ (The Jackson Laboratory, Stock No. 012930), *hGFAP-Cre* (The Jackson Laboratory, Stock No. 004600), and *Emx1-Cre* (The Jackson Laboratory, Stock No. 005628). All experiments were carried out in compliance with ethical regulations for animal research. Our study protocol was reviewed and approved by the University of Michigan Institutional Animal Care & Use Committee.

### Primary culture of mouse NPCs

Mouse NPCs were isolated from *Knl1* cKO, *Knl1*;*Trp53* dKO, and littermate control E15.5 cortices, enzymatically dissociated using Papain/Dispase/DNaseI (Worthington Biochemical), and cultured in neurobasal medium (GIBCO) supplemented with bFGF (GIBCO), B27 (GIBCO), Glutamax (Invitrogen), and penicilin/streptomycin (Invitrogen) on glass coverslips precoated with poly-l-ornithine and laminin.

### Immunohistochemistry and confocal imaging

Embryonic and neonatal brains were dissected and fixed by immersion in 4% PFA overnight at 4 °C. Mouse brains were sectioned at 70 µm with a Vibratome VT1000S (Leica). For immunostaining, brain sections were incubated in blocking solution containing 5% normal donkey serum (Jackson Immuno Research Laboratories), 1% BSA, 0.1% glycine, 0.1% lysine, and 0.3% TritonX-100 in PBS for 1 h^62^. Sections were then incubated overnight at 4 °C in primary antibodies diluted in blocking solution, washed, and incubated with the appropriate fluorescent secondary antibodies and DAPI or processed for immunohistochemistry. Stained sections were mounted with VECTASHIELD Antifade Mounting Medium (Vector Laboratories) and analyzed. Images were acquired with the Olympus confocal microscope FV1000. ImageJ and Photoshop were used for image processing. Primary antibodies: rabbit anti-SOX2 (Millipore,1:500), rat anti-SOX2 (Thermo Fisher Scientific, 1:500), rat anti-EOMES (TBR2) (Thermo Fisher Scientific, 1:500), chicken anti-RBFOX3 (NEUN) (Millipore, 1:500), rabbit anti-cleaved Caspase-3 (CC3) (Cell Signaling Technology, 1:500), mouse anti-VIM (phospho-Ser55) (Enzo Life Sciences, 1:500), rat anti-BCL11B (Abcam, 1:500), rabbit anti-CUX1 (Santa Cruz Biotechnology, 1:250), rabbit anti-KI67 (Abcam,1:250), rabbit anti-p53 (Leica Microsystems, 1:250), rabbit anti-histone H2A.X (phospho-Ser139) (Cell Signaling Technology, 1:250), rabbit anti-IBA1 (AIF1) (Wako, 1:500), rabbit anti-ADGRE1 (F4/80) (Abcam, 1:500), goat anti-POU3F2 (BRN2) (Santa Cruz Biotechnology, 1:250), rabbit anti-TBR1 (Abcam, 1:500), rabbit anti-phospho KAP1(S824) (Bethyl, 1:500), mouse anti-SATB2 (Abcam, 1:500), rat anti-CldU (Accurate Chemical and Scientific Corporation, 1:250), chicken anti-GFP (Abcam,1:500), rabbit anti-GFP (Thermo Fisher Scientific, 1:500). Secondary antibodies: Alexa Fluor 488 AffiniPure Donkey Anti-Rabbit IgG (H+L) (Jackson ImmunoResearch Labs, 1:250), Alexa Fluor 488 AffiniPure Donkey Anti-Rat IgG (H+L) (Jackson ImmunoResearch Labs, 1:250), Alexa Fluor 488 AffiniPure Donkey Anti-Mouse IgG (H+L) (Jackson ImmunoResearch Labs, 1:250), Alexa Fluor 488 AffiniPure Bovine Anti-Goat IgG (H+L) (Jackson ImmunoResearch Labs, 1:250), Alexa Fluor 594 AffiniPure Donkey Anti-Mouse IgG (H+L) (Jackson ImmunoResearch Labs, 1:250), Alexa Fluor 594 AffiniPure Donkey Anti-Rabbit IgG (H+L) (Jackson ImmunoResearch Labs, 1:250), Alexa Fluor 594 AffiniPure Donkey Anti-Rat IgG (H+L) (Jackson ImmunoResearch Labs, 1:250), Alexa Fluor 594 AffiniPure Bovine Anti-Goat IgG (H+L) (Jackson ImmunoResearch Labs, 1:250).

### RNA purification and droplet digital PCR (ddPCR)

Total RNA was isolated from freshly microdissected embryonic 15.5 cortices using Trizol and RNA Clean & Concentrator Kit. 600 ng of RNA was used as input for cDNA synthesis using SuperScript III (Invitrogen). Diluted cDNA was used for expression level analysis using ddPCR (QX200 Droplet Digital PCR System, Bio-Rad). Probe sets (PrimeTime qPCR Probe Assays) were purchased from Integrated DNA Technologies. For ddPCR, the reaction mixture was prepared with template cDNA, 2XddPCR Supermix (No dUTP) (Bio-Rad), target probes with HEX fluorescence, and control probe against house-keeping gene *Srp72* with FAM fluorescence. Oil droplets were generated with the QX200 droplet generator (Bio-Rad) and transferred to the C1000 Touch Thermal cycler (Bio-Rad) for thermocycling. Following PCR amplification, the droplets were analyzed in the QX200 droplet reader (Bio-Rad), which analyzed each droplet individually using a two-color detection system.

### Primers

Primers and probes for ddRT-PCR:

*Pvt1*:

primer1 GCCACTGCCAATGTCTGT

primer2 CACTGAAAACAAGGACCGAAAC;

probe /56-FAM/TCCAGGTAG/Zen/CCCGAGAGATGACA/3IABkFQ/

*Eda2r*:

primer1 GCATCTACCTTCACTAAGCTCA

primer2 GTTCTACCGAAAGACACGCAT

probe /56-FAM/TGCATCCCA/Zen/TGTACAAAGCAGACTCC/3IABkFQ/

*Ano3*:

primer1 CGAAGAAAACTGTCCCTCCAT

primer2 GTCTTCATGTGTCCTCTATGTGA

probe /56-FAM/AGCTGTCGT/Zen/TGAGTCTCTGCAGTG/3IABkFQ/

*Eomes*:

primer1 CCAGAACCACTTCCACGAAA

primer2 CGGCACCAAACTGAGATGA

probe /56-FAM/TCAACATAA/Zen/ACGGACTCAACCCCACC/3IABkFQ/

*Srp72*:

primer1 CTCTCCTCATCATAGTCGTCCT

primer2 CTGAAGGAGCTTTATGGACAAGT

probe /5HEX/CCAAGCACT/Zen/CATCGTAGCGTTCCA/3IABkFQ/

*Bbc3* (*Puma*):

primer1 AGAGATTGTACATGACCCTCCA

primer2 GACCTCAACGCGCAGTA

probe /56-FAM/CGGAGACAA/Zen/GAAGAGCAGCATCGA/3IABkFQ/

Genotyping primers:

Knl1_fl_200_F TCAGAATTTTGAGATGGCGCA

Knl1_WT_136_F GCTCAGTGGTATTTTGCCTAGC

Knl1_common_R AGAGTTTACCCTTTTCAACTGGA

LacZ cassette removal primers:

Knl1_lacZ_F CCTACCTTTGCCACCTGAGT

Knl1_lacZ_R GTTCCTGACCTGACTTCCCA

### CIdU, IdU, and EdU labeling

Timed-pregnancies were obtained by detection of a vaginal plug (E0.5). Timed-pregnant mice were pulsed intraperitoneally with thymidine analogs CIdU, IdU, or EdU at 0.05 mg/g of body weight. Mice were analyzed for incorporation of CIdU or IdU using specific antibodies. Incorporation of EdU was analyzed by Click reaction using fluorescent azide.

### Metaphase chromosome spread

Mouse NPCs were isolated from *Knl1* cKO, *Knl1*;*Trp53* dKO, and littermate control E14–15 embryonic cortices and cultured in Neurobasal Medium (GIBCO) supplemented with bFGF (GIBCO), B27 (GIBCO), Glutamax (Invitrogen), and Penicilin/Streptomycin (Invitrogen). Cells were cultured in less than 36 h, and treated with colcemid (10 ng/ml) and MG132 (10 μm) for 3 h. Cells were then subjected to hypotonic swelling (by incubating in 0.56% KCl) for 15 min and fixed in Carnoy’s fixative (75% methanol, 25% acetic acid). Chromosome spreads were prepared on clean, dry slides.

### Comet assay

Single cell gel electrophoresis comet assay was performed using the SCGE assay Kit (Enzo Life Sciences). E14.5 embryonic cortices were dissected from *Knl1* cKO, *Knl1*;*Trp53* dKO, and littermate control, dissociated into single cells, and resuspended in pre-chilled PBS. Cells were mixed with low melting point agarose at a volume ratio of 1:50 and transferred in 100 μl aliquots onto pre-warmed slides. Slides were immersed in pre-chilled lysis solution for at least 1 h and placed in pre-chilled alkaline solution for 1 h. Electrophoresis was performed at 22 V in the TBE buffer for 30 min. Comets were stained with CYGREEN dye for 30 min and imaged.

### Anaphase analysis

NPCs were cultured on coverslips and pulsed with EdU for 12 h. Cells were washed with PBS and fixed with 4% PFA for 30 min at room temperature and washed three times with PBS for about 20 min. Cells were then subjected to Click reaction using fluorescent azide and immunostained with γH2AX primary antibody as described above. DNA was counterstained with DAPI diluted in PBS for 5 min. Coverslips were mounted with VECTASHIELD Antifade Mounting Medium (Vector Laboratories).

### ClickSeq

RNA-seq libraries were generated using ClickSeq^36^. Total RNA was extracted using Trizol (Invitrogen), treated with DNaseI, and cleaned and concentrated using the RNA Clean and Concentrator Kit (Zymo Research). Ribosomal RNA was removed using NEBNext rRNA Depletion Kit (NEB). ERCC RNA spike-in standards (Thermo Fisher Scientific) were used to assess library quality. Reverse transcription was carried out with SuperScript II (Invitrogen) using 1:30 5 mM AzdNTP:dNTP and 3′Genomic Adapter-6N RT primer (GTGACTGGAGTTCAGACGTGTGCTCTTCCGATCTNNNNNN). The reaction was then treated with RNaseH to remove RNA template and cleaned using DNA Clean and Concentrator Kit (Zymo Research). The azido-terminated cDNA was diluted with DMSO and click adaptor (/5Hexynyl/NNNNNNNNAGATCGGAAGAGCGTCGTGTAGGGAAAGAGTGTAGATCTCGGTGGTCGCCGTATCATT). The click reaction was initiated by adding Cu2+ (Lumiprobe) in the presence of vitamin C. The resulting click ligation was purified using DNA Clean and Concentrator Kit (Zymo).

The final amplification step was carried out using 2x One Taq Hot Start Mastermix (NEB), using Illumina universal primer (AATGATACGGCGACCACCGAG) and Illumina indexing primer (CAAGCAGAAGACGGCATACGAGATNNNNNNGTGACTGGAGTTCAGACGTGT). The PCR reaction was cleaned using Ampure XP magnetic beads (Beckman) at 1.25× ratio, which size-selects for product size above 200 bp. The resulting library was analyzed using TapeStation (Agilent) to check for proper size distribution and quality of DNA. Sequencing was performed on the Illumina NextSeq 550 platform (75 cycles, high output) at the University of Michigan sequencing core.

### RNA sequencing data analysis

RNA-seq data were subject to quality control check using FastQC v0.11.5 (https://www.bioinformatics.babraham.ac.uk/projects/download.html#fastqc). Adapter trimming was performed using cutadapt version 1.13 (http://cutadapt.readthedocs.io/en/stable/guide.html). Processed reads were aligned to the GENCODE GRCm38/mm10 reference genome (https://www.gencodegenes.org/mouse_releases/current.html) with STAR (v2.5.2a) and deduplicated according to UMI using UMI-tools (v0.5.3)^63,64^. Read counts were obtained with htseq-count (v0.6.1p1) with intersection-nonempty mode^65^. Differential expression was determined with edgeR^37^. The *p*-value was calculated with likelihood ratio tests and the adjusted *p*-value for multiple testing was calculated using the Benjamini–Hochberg procedure, which controls FDR. All RNA-seq data have been deposited in the GEO database (GSE114956).

### Image data analysis

Image data quantification was performed using ImageJ or ImageJ (Fiji) with age-matched and spatially matched brain sections from mutants and control cortices.

### Statistical analysis

*Knl1* mutant and control group comparison statistical analysis were performed using GraphPad Prism 7 (GraphPad Software) using unpaired two-tailed Student’s *t*-test. A *p*-value of <0.05 was considered statistically significant. ERCC quantification performed by Salmon using ERCC Mix 1 sequences^66^. The mean and 95% confidence interval were calculated by aggregating all measurements of ERCC sequences of equal concentration across all samples. Gene lists associated with p53 activation and microglia were matched by gene symbol. *Knl1* cKO and dKO intersectional comparison was performed using a paired two-tailed Student’s *t*-test.

### Reporting summary

Further information on research design is available in the Nature Research Reporting Summary linked to this article.

## Supplementary information

Supplementary Information

Peer Review

Reporting Summary

Source Data

## Data Availability

The RNA-seq data that support the findings of this study have been deposited to NCBI GEO with the accession number GSE114956. The source data underlying Figs. 1–8 and Supplementary Figs. 1–6 are provided in a Source Data file.
