# Supplementary material for: Robust elimination of genome-damaged cells safeguards against brain somatic aneuploidy following Knl1 deletion
Source: Nat Commun. 2019 Jun 13;10:2588. doi: 10.1038/s41467-019-10411-w (PMC6565622; doi:10.1038/s41467-019-10411-w)
Supplement: Supplementary file 1 — Supplementary Information [file 41467_2019_10411_MOESM1_ESM.pdf]

Supplementary Information

Robust elimination of genome-damaged cells safeguards against brain somatic aneuploidy following *Knl1* deletion

Lei Shi[1,2], Adel Qalieh[1,2], Mandy M. Lam[1,2], Jason M. Keil[1,2,3], Kenneth Y. Kwan[1,2,*]

1. Molecular & Behavioral Neuroscience Institute (MBNI), University of Michigan, Ann Arbor, MI, 48109, USA.
2. Department of Human Genetics, University of Michigan, Ann Arbor, MI, 48109, USA.
3. Medical Scientist Training Program, University of Michigan, Ann Arbor, MI, 48109, USA.

*Correspondence should be addressed to K.Y.K. (kykwan@umich.edu)

Supplementary Fig. 1

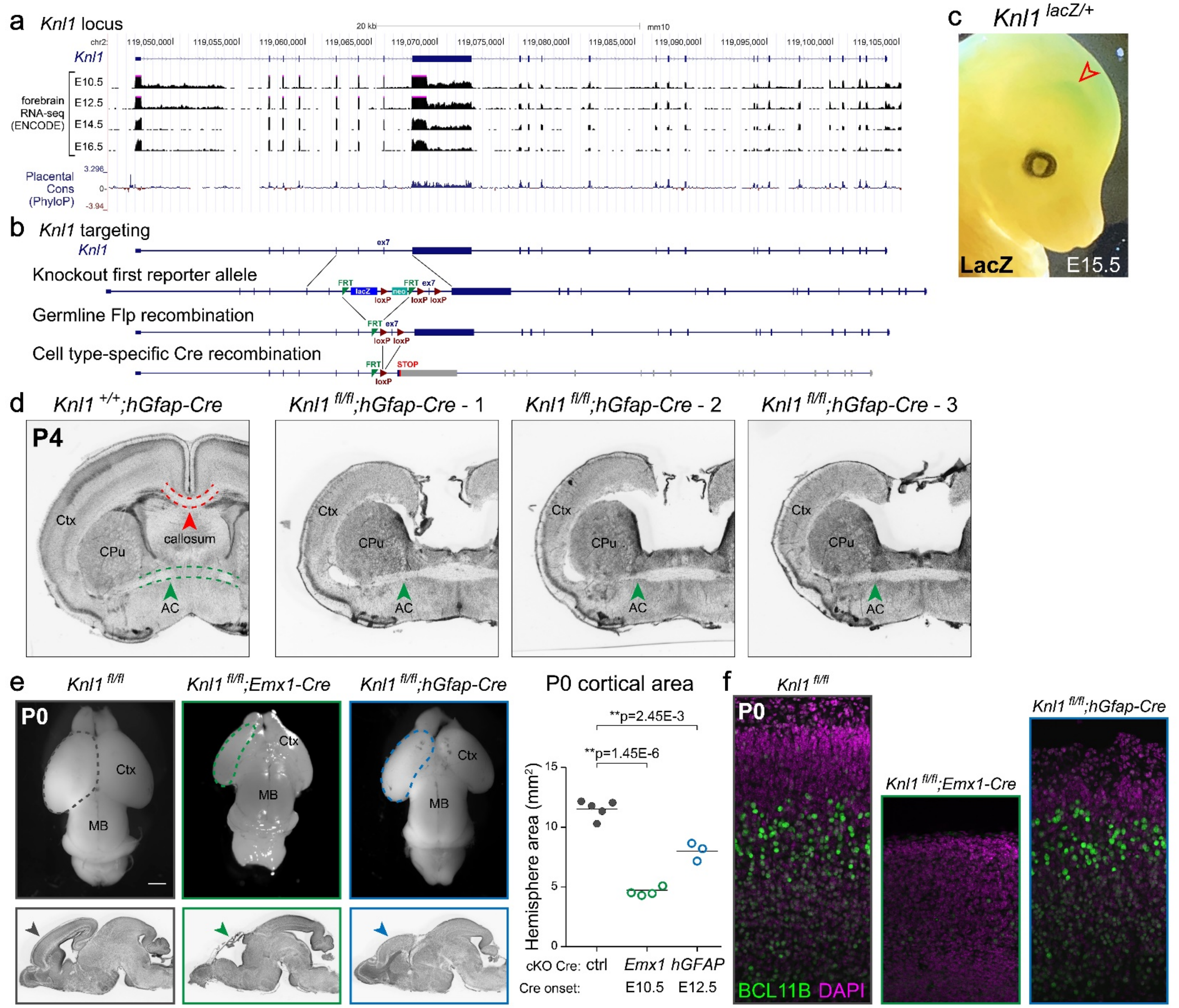

a The mouse *Knl1* locus. ENCODE bulk RNA-seq of embryonic forebrain revealed expression of *Knl1* throughout the neurogenic period. **b** *Knl1* locus targeting and generation of lacZ reporter and conditional alleles. **c** Whole mount of E15.5 embryo showed lacZ staining in embryonic cortex (arrowhead). **d** At P4, *Knl1* cKO brains exhibited agenesis of corpus callosum (red arrowhead). Anterior commissure (AC, green arrowheads) was not affected. **e**, **f** Developmentally earlier deletion of *Knl1* using *Emx1-Cre* led to microcephaly more severe than *hGFAP-Cre* (cortical area, mean, two-tailed unpaired *t* test, ctrl: *n*=5, *Emx1-Cre* cKO: *n*=5, *hGFAP-Cre* cKO: *n*=3 animals). In addition to loss of upper layer neurons, BCL11B+ (green) deep layer neurons were also decreased in *Emx1-Cre* cKO.

Supplementary Fig. 2

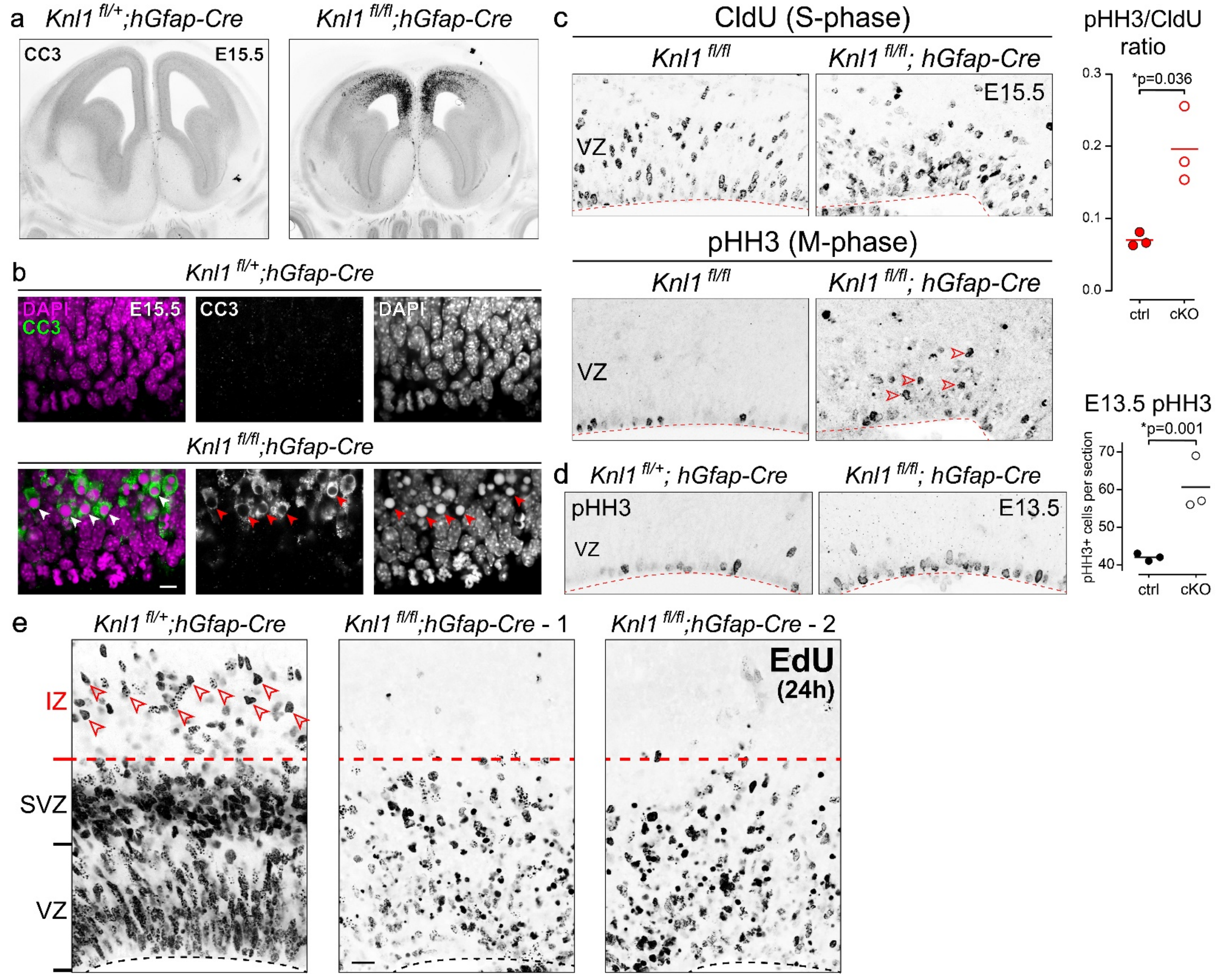

a CC3 immunostaining showed extensive apoptosis in E15.5 cKO cortex. **b** Pyknotic cells (arrowheads) revealed by DAPI staining (magenta) were immunopositive for CC3 (green), indicating that they were apoptotic (scale bar: 20μm). **c** Analysis of S-phase cells labeled by a 4-hour pulse of CldU and M-phase cells labeled by pHH3 revealed a significant increase in M-phase cells in the E15.5 cKO cortex, suggesting incompletion or delayed completion of mitosis (mean, two-tailed unpaired *t* test, *n*=3 animals). Some pHH3+ cells were abventricular (open arrowheads). **d** The increase in pHH3 staining was present at E13.5, preceding widespread apoptosis (mean, two-tailed unpaired *t* test, *n*=3 animals). **e** Analysis of E16.5 cortex following a 24-hour pulse of EdU. EdU+ migrating neurons (red arrowheads) were present in the IZ of ctrl but largely absent from cKO (scale bar: 20μm).

Supplementary Fig. 3

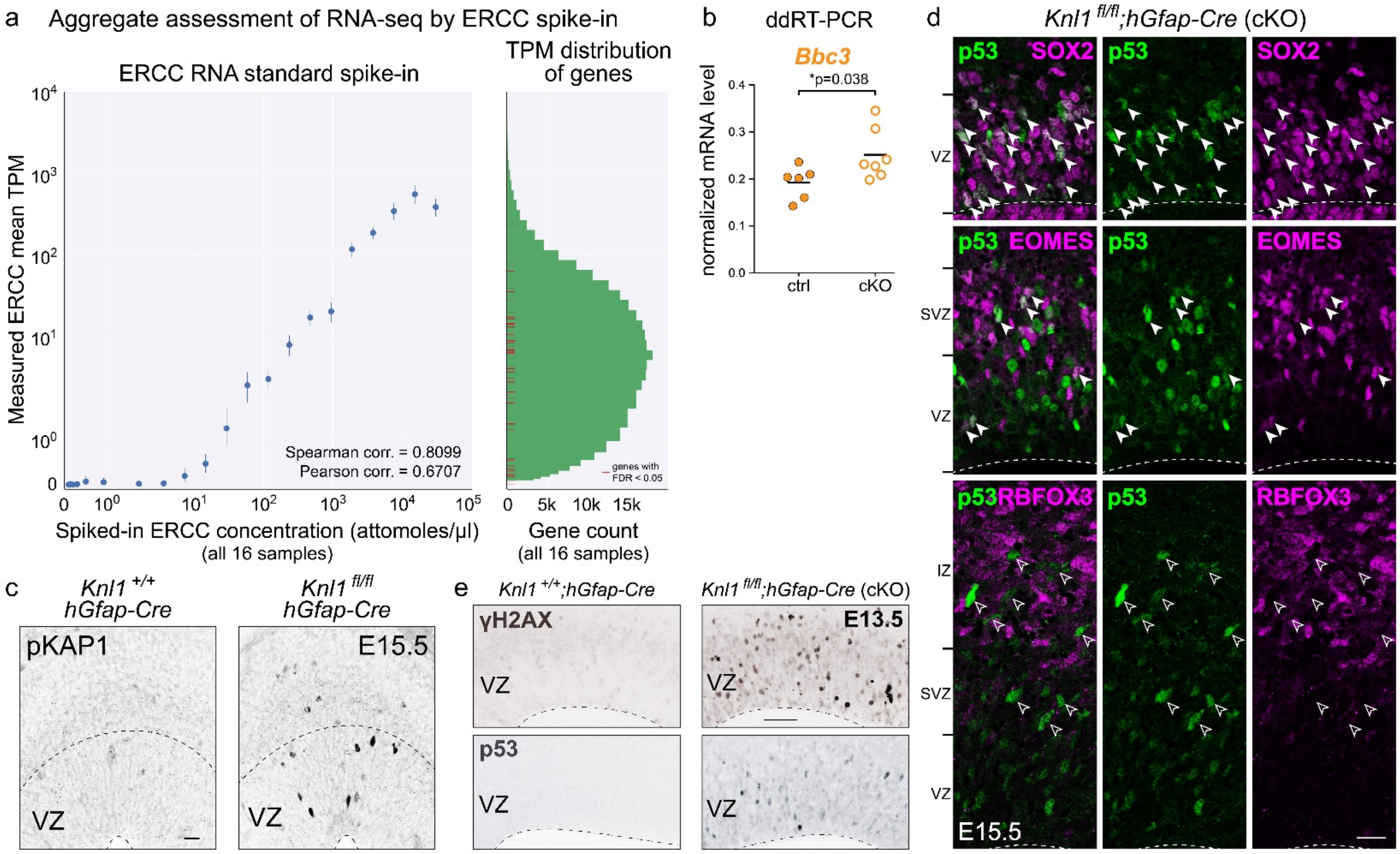

a Aggregate assessment of ERCC spike-in standards in UMI RNA-seq revealed excellent correlation over a broad dynamic range. The vast majority of genes from all 16 experimental samples were within the dynamic range of UMI RNA-seq. TPM, transcripts per million. **b** ddRT-PCR of p53 target gene *Bbc3* (*Puma*) in cKO compared to ctrl (mean, two-tailed unpaired *t* test, ctrl: *n*=6, cKO: *n*=7 animals). **C** Immunostaining revealed VZ cells labeled by heterochromatic DSB marker pKAP1 in E15.5 cKO (scale bar: 20μm). **d** Co-immunostaining in E15.5 cKO revealed p53 activation (green) in SOX2+ and EOMES+ NPCs (solid arrowheads), but largely not in RBFOX3+ neurons (open arrowheads, scale bar: 20μm). **e** DNA damage markers, γH2AX and p53, were widespread in cKO VZ at E13.5, when the majority of cells in the cortical wall were NPCs, and preceding massive apoptosis in cKO (scale bar: 50μm).

Supplementary Fig. 4

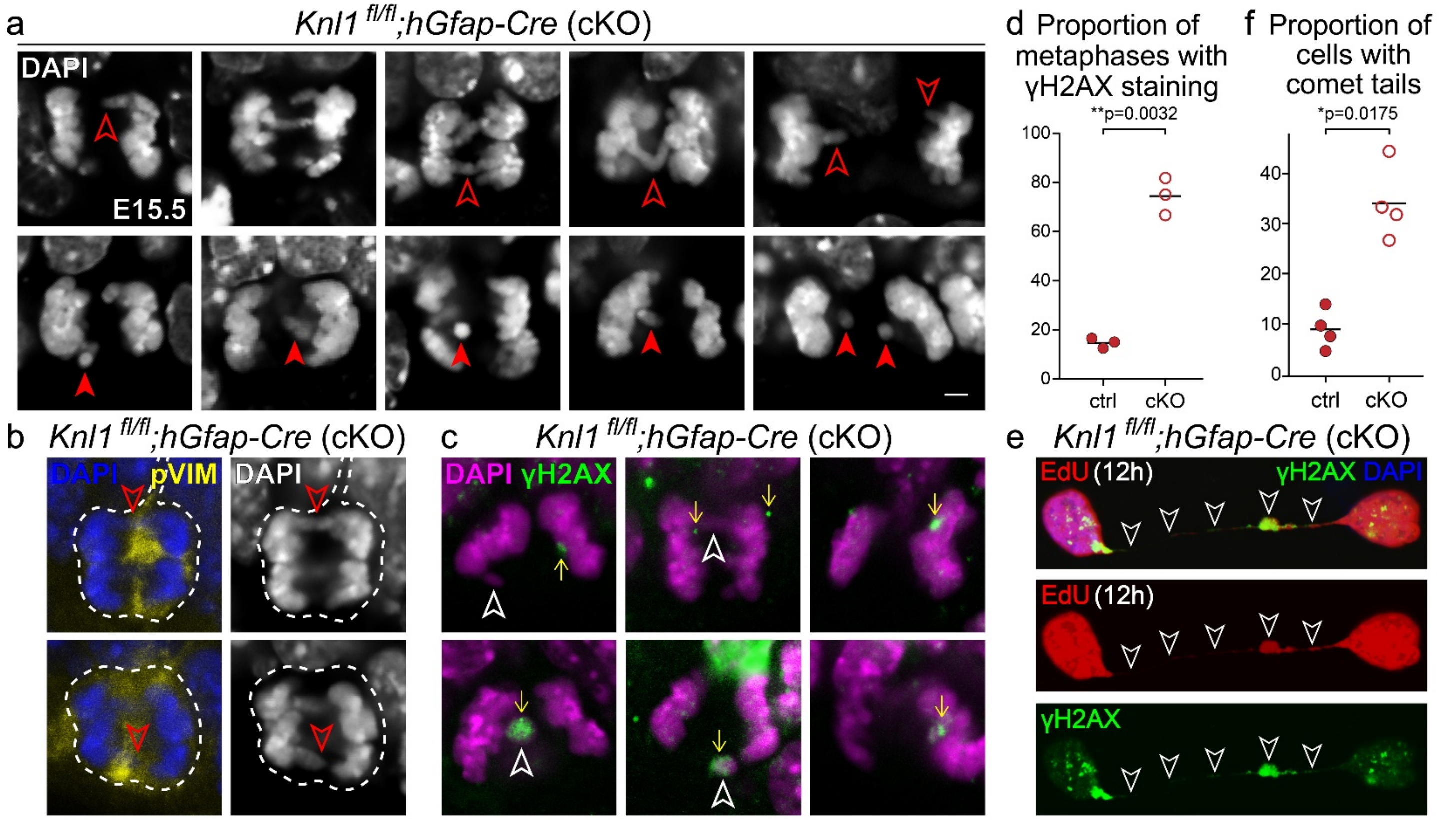

a Additional examples of frequent lagging and bridged chromosomes (open arrowheads), and micronuclei (solid arrowheads) in anaphase NPCs in E15.5 cKO cortex (scale bar: 2μm). **b** Lagging chromosomes (open arrowheads) were present in the midzone labeled by phospho-Vimentin (pVIM, yellow). **c** Missegregated chromosomes and micronuclei (open arrowheads) in E15.5 cKO cortex were immunopositive for DNA damage marker γH2AX (green, and arrows). **d** The proportion of metaphases with γH2AX foci was significantly increased in cKO compared to ctrl (mean, two-tailed unpaired *t* test, *n*=3 animals). **e** Dissociated E15.5 cortical NPC culture after 2 days in vitro (DIV) and a 12-hour pulse of EdU (red). NPCs cultured from cKO were characterized by DNA bridges (open arrowheads) immunopositive for γH2AX (green). **f** Single cell electrophoresis comet assay showed a significant increase in comet tailing in cKO compared to ctrl (mean, two-tailed unpaired *t* test, *n*=4 animals).

Supplementary Fig. 5

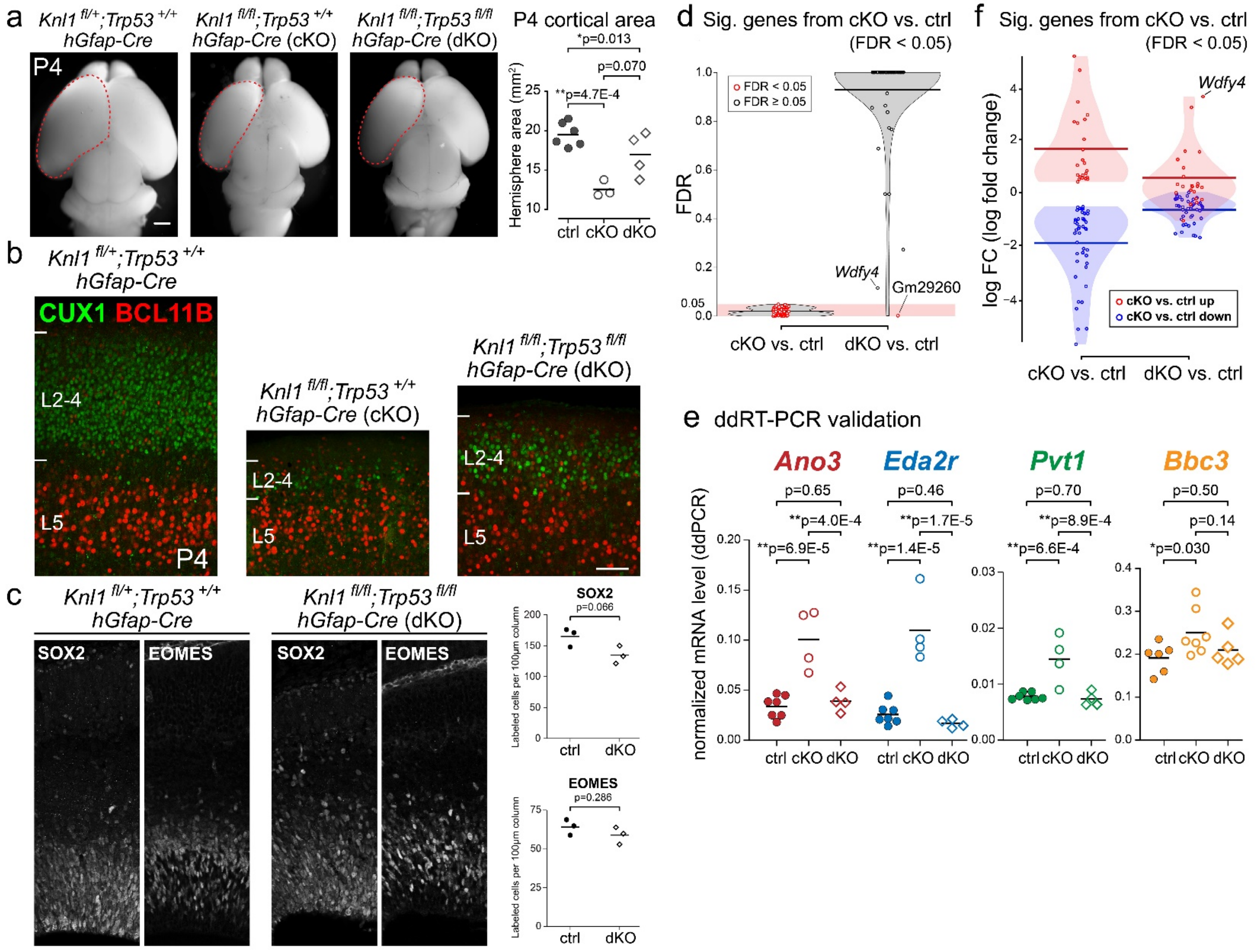

a Dorsal view of ctrl, cKO, and dKO P4 brain (scale bar: 1mm). The significant cortical area loss in cKO compared to littermate control (ctrl) was partially ameliorated in dKO (mean, ANOVA with post hoc *t* test, ctrl: *n*=6, cKO: *n*=3, dKO: *n*=4 animals). **b** Cortical layer marker analysis at P4 revealed no change in BCL11B+ (L5, red) neurons (scale bar: 100µm). The reduction in CUX1+ (L2-4, green) neurons in cKO compared to ctrl was partially ameliorated in dKO. **c** NPC marker analysis of E15.5 cortex revealed no significant decrease in SOX2+ apical progenitors or EOMES+ intermediate progenitors in dKO compared to ctrl (mean, two-tailed unpaired *t* test, *n*=3 animals). **d** Violin plot of all differentially expressed genes from cKO compared to ctrl, and corresponding FDR from dKO compared to ctrl. The majority of differentially expressed genes from cKO compared to ctrl were not differentially expressed in dKO compared to ctrl. **e** Analysis by ddRT-PCR validated that cKO expression changes in p53-dependent genes *Ano3, Eda2r*, and *Pvt1* were corrected to levels similar to ctrl in dKO (mean, ANOVA with post hoc *t* test, ctrl: *n*=7, cKO: *n*=4, dKO: *n*=4 animals). *Bbc3* (*Puma*) expression was not significantly changed in dKO compared to ctrl (ctrl: *n*=6, cKO: *n*=7, dKO: *n*=5 animals). **f** Violin plots of all significantly upregulated (red) and downregulated (blue) genes from cKO compared to ctrl, and corresponding log fold change (log FC) from dKO compared to ctrl. The majority of up- and downregulated genes from cKO compared to ctrl were not differentially expressed in dKO compared to ctrl (log FC shifted towards 0).

Supplementary Fig. 6

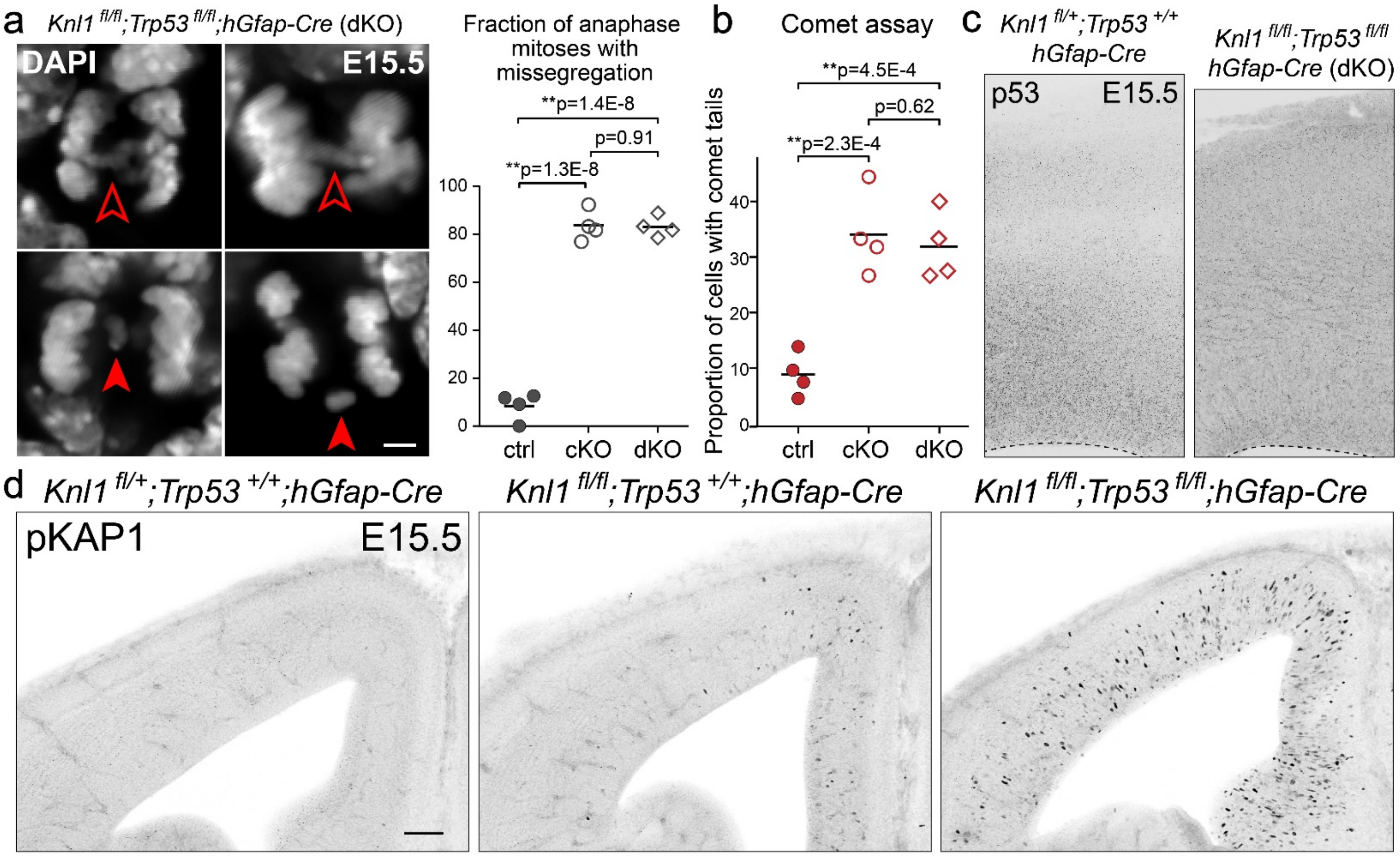

a Additional examples of lagging and bridged chromosomes (open arrowheads), and micronuclei (solid arrowheads) in anaphase NPCs in E15.5 dKO cortex (scale bar: 2μm). The fraction of anaphase mitoses with missegregation was significantly increased in dKO similar to cKO (mean, ANOVA with post hoc *t* test, *n*=4 animals). **b** Single cell electrophoresis comet assay showed a significant increase in comet tailing in dKO compared to ctrl (mean, ANOVA with post hoc *t* test, *n*=4 animals). **c** Immunostaining revealed no activation of p53 in E15.5 dKO cortex. **d** Immunostaining of E15.5 cortex revealed inefficient clearance of cells labeled by heterochromatic DSB marker pKAP1 in dKO (scale bar: 100μm).

Supplementary Fig. 7

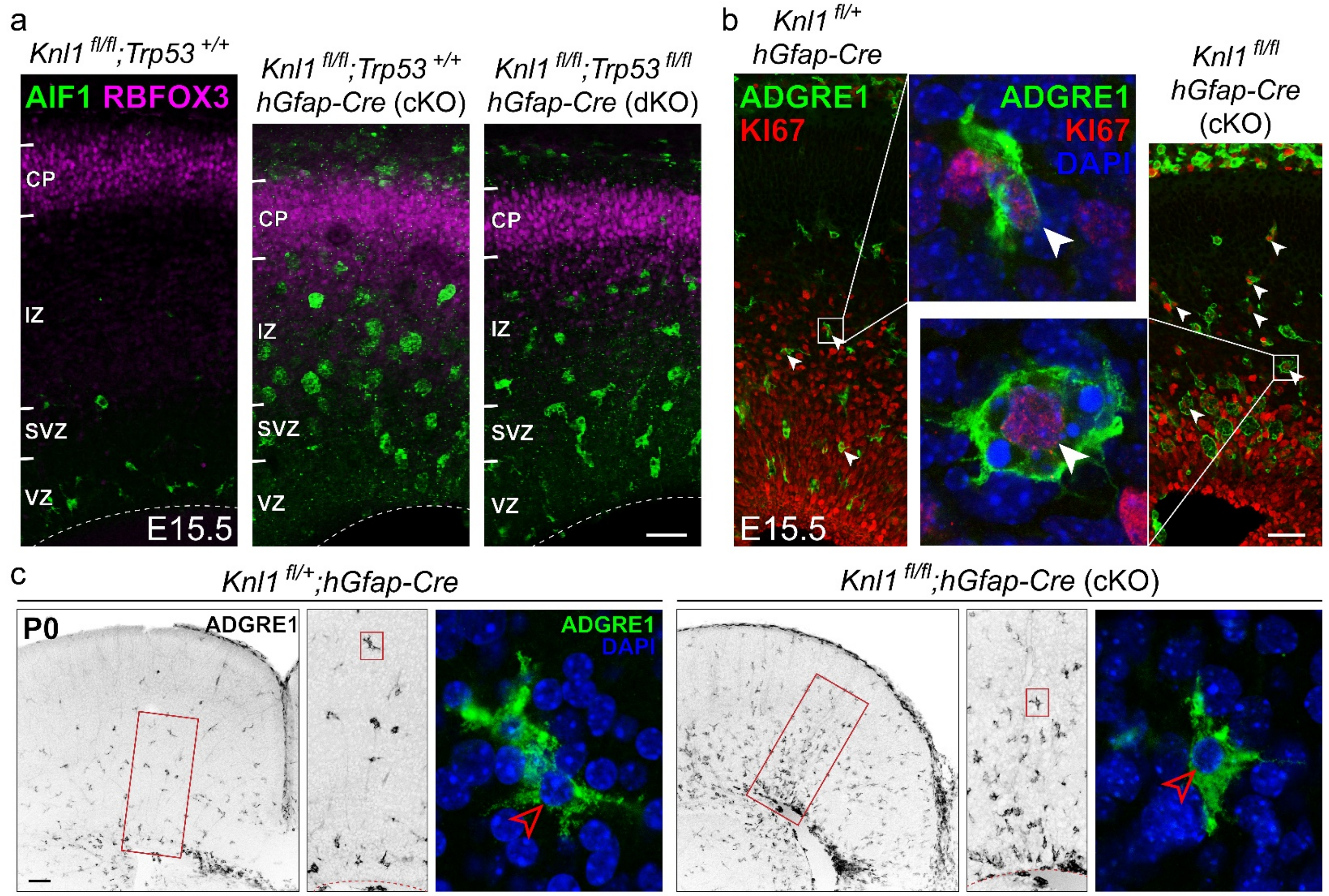

a AIF1 (IBA1, green) immunostaining revealed morphologic activation of microglia in E15.5 cKO and dKO cortex (scale bar: 50µm). **b** ADGRE1+ (green) microglia were immunopositive for the proliferation marker, KI67 (red), in both E15.5 ctrl and dKO cortex (scale bar: 50µm). **c** By birth (P0), ADGRE1+ microglia have returned to resting morphology and were mononuclear (open arrowheads) in the cKO (scale bar: 100µm).

Supplementary Table 1

All significantly up- and down-regulated transcripts by UMI-RNA-seq (cKO vs. ctrl, FDR < 0.05)

Ensembl Mouse Gene ID	Gene symbol	logFC	logCPM	LR	PValue	FDR	p53 target	microglia	interneuron	*hGFAP* lineage
ENSMUSG00000074968.11	*Ano3*	2.08	4.81	138.85	4.74E-32	2.46E-27				
ENSMUSG00000097039.8	*Pvt1*	1.15	5.23	58.76	1.79E-14	4.63E-10				
ENSMUSG00000034457.10	*Eda2r*	2.70	2.66	56.39	5.93E-14	1.03E-09				
ENSMUSG00000004085.14	*Map3k20*	0.76	4.77	26.54	2.59E-07	0.0015				
ENSMUSG00000046178.3	*Nxph1*	0.61	9.09	22.99	1.63E-06	0.0056				
ENSMUSG00000021185.16	*Dglucy*	1.27	2.76	22.37	2.25E-06	0.0065				
ENSMUSG00000023067.13	*Cdkn1a*	1.68	2.22	21.72	3.15E-06	0.0080				
ENSMUSG00000102813.1	NA	5.29	0.52	21.07	4.42E-06	0.0096				
ENSMUSG00000106379.1	*Lhfpl3*	0.55	7.47	20.49	5.99E-06	0.0111				
ENSMUSG00000002699.13	*Lcp2*	2.75	1.08	20.57	5.76E-06	0.0111				
ENSMUSG00000070880.10	*Gad1*	0.58	6.76	20.12	7.28E-06	0.0126				
ENSMUSG00000036264.9	*Fstl4*	0.60	5.75	19.96	7.89E-06	0.0128				
ENSMUSG00000085912.1	*Trp53cor1*	3.01	0.67	19.07	1.26E-05	0.0170				
ENSMUSG00000020326.7	*Ccng1*	0.85	3.77	18.22	1.97E-05	0.0237				
ENSMUSG00000092247.2	NA	4.75	0.20	18.01	2.20E-05	0.0259				
ENSMUSG00000032259.8	*Drd2*	1.06	3.16	17.72	2.56E-05	0.0288				
ENSMUSG00000097311.7	NA	0.53	8.94	17.16	3.44E-05	0.0339				
ENSMUSG00000086665.1	NA	2.84	0.85	17.18	3.39E-05	0.0339				
ENSMUSG00000068859.5	*Sp9*	0.69	4.54	16.89	3.96E-05	0.0367				
ENSMUSG00000037946.11	*Fgd3*	0.68	4.61	16.83	4.09E-05	0.0373				
ENSMUSG00000021338.17	*Carmil1*	0.43	7.02	16.69	4.39E-05	0.0375				
ENSMUSG00000062209.15	*Erbb4*	0.53	10.53	16.71	4.36E-05	0.0375				
ENSMUSG00000051506.16	*Wdfy4*	3.52	0.50	16.70	4.37E-05	0.0375				

Ensembl Mouse Gene ID	Gene symbol	logFC	logCPM	LR	PValue	FDR	p53 target	microglia	interneuron	*hGFAP* lineage
ENSMUSG00000001870.15	*Ltbp1*	-1.27	6.17	41.28	1.32E-10	1.71E-06				
ENSMUSG00000032446.14	*Eomes*	-1.01	5.39	31.31	2.20E-08	0.0002				
ENSMUSG00000096403.2	NA	-3.47	0.64	29.43	5.80E-08	0.0005				
ENSMUSG00000082536.1	NA	-2.23	2.16	28.10	1.15E-07	0.0009				
ENSMUSG00000056904.3	NA	-2.62	1.36	27.13	1.90E-07	0.0012				
ENSMUSG00000031273.16	*Col4a6*	-1.35	2.55	25.78	3.82E-07	0.0018				
ENSMUSG00000033377.14	*Palmd*	-0.73	4.78	25.87	3.65E-07	0.0018				
ENSMUSG00000102189.1	NA	-1.32	7.18	25.38	4.70E-07	0.0020				
ENSMUSG00000030022.14	*Adamts9*	-0.96	3.49	24.91	6.01E-07	0.0024				
ENSMUSG00000061808.3	*Ttr*	-5.61	2.65	24.54	7.26E-07	0.0027				
ENSMUSG00000100341.1	NA	-2.08	3.88	22.66	1.93E-06	0.0063				
ENSMUSG00000028640.11	*Tfap2c*	-0.97	3.59	22.46	2.14E-06	0.0065				
ENSMUSG00000092702.2	NA	-2.26	2.85	22.08	2.62E-06	0.0072				
ENSMUSG00000053441.4	*Adamts19*	-1.15	3.25	21.68	3.22E-06	0.0080				
ENSMUSG00000030237.14	*Slco1a4*	-5.06	-0.18	21.50	3.54E-06	0.0084				
ENSMUSG00000053508.7	*BC048502*	-1.50	2.87	21.41	3.71E-06	0.0084				
ENSMUSG00000083563.1	NA	-2.54	2.19	20.53	5.87E-06	0.0111				
ENSMUSG00000031274.16	*Col4a5*	-1.09	3.61	20.67	5.46E-06	0.0111				
ENSMUSG00000086155.7	*9430041J12Rik*	-1.10	3.26	20.12	7.28E-06	0.0126				
ENSMUSG00000027217.13	*Tspan18*	-0.59	6.38	19.98	7.84E-06	0.0128				
ENSMUSG00000001911.16	*Nfix*	-0.68	8.67	19.76	8.79E-06	0.0138				
ENSMUSG00000101795.2	NA	-1.84	0.97	19.56	9.74E-06	0.0149				
ENSMUSG00000087413.1	NA	-0.78	6.25	19.46	1.03E-05	0.0153				
ENSMUSG00000055653.13	*Gpc3*	-1.02	3.32	19.37	1.07E-05	0.0155				
ENSMUSG00000075395.10	*A630010A05Rik*	-5.04	-0.33	19.18	1.19E-05	0.0167				
ENSMUSG00000047497.9	*Adamts12*	-1.25	2.39	19.04	1.28E-05	0.0170				
ENSMUSG00000059195.10	NA	-2.12	1.72	18.83	1.43E-05	0.0182				
ENSMUSG00000040183.13	*Ankrd6*	-0.60	6.25	18.79	1.46E-05	0.0182				
ENSMUSG00000029769.16	*Ccdc136*	-0.54	5.83	18.77	1.47E-05	0.0182				
ENSMUSG00000068082.12	*Grxcr1*	-2.76	1.11	17.74	2.53E-05	0.0288				
ENSMUSG00000004892.13	*Bcan*	-1.33	3.17	17.69	2.61E-05	0.0288				
ENSMUSG00000013584.5	*Aldh1a2*	-4.30	-0.17	17.54	2.81E-05	0.0304				
ENSMUSG00000036617.16	*Etl4*	-0.52	7.83	17.49	2.89E-05	0.0306				
ENSMUSG00000064397.1	NA	-2.22	4.86	17.30	3.20E-05	0.0332				
ENSMUSG00000100832.1	Gm29260	-0.60	8.42	17.14	3.46E-05	0.0339				
ENSMUSG00000106248.1	NA	-3.12	0.15	17.08	3.58E-05	0.0344				
ENSMUSG00000082454.3	NA	-1.38	2.84	17.04	3.66E-05	0.0345				
ENSMUSG00000084901.6	Gm13266	-4.04	-0.48	16.69	4.40E-05	0.0375				
ENSMUSG00000016028.9	*Celsr1*	-0.78	6.82	16.56	4.73E-05	0.0396				
ENSMUSG00000110250.1	NA	-4.05	-0.48	16.49	4.89E-05	0.0403				
ENSMUSG00000033726.8	*Emx1*	-0.76	4.49	16.39	5.14E-05	0.0417				
ENSMUSG00000027967.8	*Neurog2*	-1.00	4.36	16.30	5.40E-05	0.0432				
ENSMUSG00000023411.11	*Nfatc4*	-1.50	2.19	16.24	5.57E-05	0.0438				
ENSMUSG00000082284.1	NA	-2.33	0.71	16.12	5.94E-05	0.0460				
ENSMUSG00000033083.16	*Tbc1d4*	-0.65	5.13	16.02	6.26E-05	0.0478				

Supplementary Table 2

All significantly up- and down-regulated transcripts by UMI-RNA-seq (dKO vs. ctrl, FDR < 0.05)

Ensembl Mouse Gene ID	Gene symbol	logFC	logCPM	LR	PValue	FDR	p53 target	microglia	interneuron	*hGFAP* lineage
ENSMUSG00000043336.14	*Filip1l*	4.77	4.89	54.36	1.67E-13	8.65E-09				
ENSMUSG00000065922.1	NA	5.89	3.60	46.09	1.13E-11	2.94E-07				
ENSMUSG00000088595.1	NA	5.63	3.47	42.86	5.88E-11	1.02E-06				
ENSMUSG00000106106.2	NA	4.81	5.64	42.23	8.10E-11	1.05E-06				
ENSMUSG00000035202.8	*Lars2*	3.23	8.08	36.71	1.37E-09	1.43E-05				
ENSMUSG00000086324.8	NA	3.65	6.02	33.95	5.66E-09	4.90E-05				
ENSMUSG00000005087.17	*Cd44*	3.69	4.69	31.48	2.01E-08	0.0001				
ENSMUSG00000096780.7	NA	1.34	3.59	30.25	3.80E-08	0.0002				
ENSMUSG00000021665.8	*Hexb*	3.49	6.53	28.25	1.06E-07	0.0006				
ENSMUSG00000102349.1	NA	3.09	3.37	28.19	1.10E-07	0.0006				
ENSMUSG00000098178.1	NA	3.11	11.71	28.01	1.20E-07	0.0006				
ENSMUSG00000050377.7	*Il31ra*	3.42	4.94	26.28	2.95E-07	0.0012				
ENSMUSG00000100832.1	Gm29260	-0.63	8.40	25.69	4.00E-07	0.0015				
ENSMUSG00000022748.7	*Cmss1*	2.91	10.07	25.19	5.19E-07	0.0018				
ENSMUSG00000097625.1	NA	2.54	4.85	23.51	1.24E-06	0.0040				
ENSMUSG00000084998.1	NA	2.87	3.20	23.26	1.42E-06	0.0043				
ENSMUSG00000086805.9	NA	3.97	1.39	23.03	1.59E-06	0.0046				
ENSMUSG00000090625.1	NA	3.38	3.03	22.47	2.13E-06	0.0058				
ENSMUSG00000079564.3	Gm11149	3.97	2.50	21.97	2.77E-06	0.0072				
ENSMUSG00000085560.2	NA	3.93	2.04	20.47	6.06E-06	0.0150				
ENSMUSG00000015843.10	*Rxrg*	1.37	3.64	20.11	7.32E-06	0.0173				
ENSMUSG00000047419.5	*Cmya5*	1.51	2.48	20.00	7.73E-06	0.0174				
ENSMUSG00000069917.7	*Hba-a2*	1.98	2.02	19.50	1.00E-05	0.0217				
ENSMUSG00000097267.1	*1700109K24Rik*	4.33	1.89	19.24	1.15E-05	0.0239				
ENSMUSG00000091390.1	NA	4.00	1.50	17.96	2.25E-05	0.0450				

Supplementary Table 3

All significantly up- and down-regulated genes from cKO vs. ctrl (FDR < 0.05)

		**cKO vs. ctrl**		**dKO vs. ctrl**					
Ensembl Mouse Gene ID	Gene symbol	logFC	FDR	logFC	FDR	p53 target	microglia	interneuron	*hGFAP* lineage
ENSMUSG00000074968.11	*Ano3*	2.08	2.46E-27	0.63	1.0000				
ENSMUSG00000097039.8	*Pvt1*	1.15	4.63E-10	0.26	1.0000				
ENSMUSG00000034457.10	*Eda2r*	2.70	1.03E-09	-0.38	1.0000				
ENSMUSG00000004085.14	*Map3k20*	0.76	0.0015	-0.22	1.0000				
ENSMUSG00000046178.3	*Nxph1*	0.61	0.0056	0.24	1.0000				
ENSMUSG00000021185.16	*Dglucy*	1.27	0.0065	0.00	1.0000				
ENSMUSG00000023067.13	*Cdkn1a*	1.68	0.0080	-0.26	1.0000				
ENSMUSG00000106379.1	*Lhfpl3*	0.55	0.0111	0.25	1.0000				
ENSMUSG00000002699.13	*Lcp2*	2.75	0.0111	1.61	1.0000				
ENSMUSG00000070880.10	*Gad1*	0.58	0.0126	0.10	1.0000				
ENSMUSG00000036264.9	*Fstl4*	0.60	0.0128	0.25	1.0000				
ENSMUSG00000085912.1	*Trp53cor1*	3.01	0.0170	1.59	1.0000				
ENSMUSG00000020326.7	*Ccng1*	0.85	0.0237	-0.45	1.0000				
ENSMUSG00000032259.8	*Drd2*	1.06	0.0288	0.97	0.5006				
ENSMUSG00000068859.5	*Sp9*	0.69	0.0367	0.33	1.0000				
ENSMUSG00000037946.11	*Fgd3*	0.68	0.0373	0.27	1.0000				
ENSMUSG00000021338.17	*Carmil1*	0.43	0.0375	0.35	0.7657				
ENSMUSG00000062209.15	*Erbb4*	0.53	0.0375	0.04	1.0000				
ENSMUSG00000051506.16	*Wdfy4*	3.52	0.0375	3.72	0.1149				

		**cKO vs. ctrl**		**dKO vs. ctrl**					
Ensembl Mouse Gene Stable ID	Gene symbol	logFC	FDR	logFC	FDR	p53 target	microglia	interneuron	*hGFAP* lineage
ENSMUSG00000001870.15	*Ltbp1*	-1.27	1.71E-06	-0.48	1.0000				
ENSMUSG00000032446.14	*Eomes*	-1.01	0.0002	-0.51	0.9137				
ENSMUSG00000031273.16	*Col4a6*	-1.35	0.0018	-0.38	1.0000				
ENSMUSG00000033377.14	*Palmd*	-0.73	0.0018	-0.23	1.0000				
ENSMUSG00000030022.14	*Adamts9*	-0.96	0.0024	-0.25	1.0000				
ENSMUSG00000061808.3	*Ttr*	-5.61	0.0027	-0.55	1.0000				
ENSMUSG00000028640.11	*Tfap2c*	-0.97	0.0065	-0.59	1.0000				
ENSMUSG00000053441.4	*Adamts19*	-1.15	0.0080	-0.60	1.0000				
ENSMUSG00000030237.14	*Slco1a4*	-5.06	0.0084	-1.23	1.0000				
ENSMUSG00000053508.7	*BC048502*	-1.50	0.0084	-0.85	1.0000				
ENSMUSG00000031274.16	*Col4a5*	-1.09	0.0111	-0.42	1.0000				
ENSMUSG00000086155.7	*9430041J12Rik*	-1.10	0.0126	-0.87	0.7719				
ENSMUSG00000027217.13	*Tspan18*	-0.59	0.0128	-0.19	1.0000				
ENSMUSG00000001911.16	*Nfix*	-0.68	0.0138	-0.25	1.0000				
ENSMUSG00000055653.13	*Gpc3*	-1.02	0.0155	-0.12	1.0000				
ENSMUSG00000075395.10	*A630010A05Rik*	-5.04	0.0167	-1.59	1.0000				
ENSMUSG00000047497.9	*Adamts12*	-1.25	0.0170	0.01	1.0000				
ENSMUSG00000040183.13	*Ankrd6*	-0.60	0.0182	-0.47	0.5003				
ENSMUSG00000029769.16	*Ccdc136*	-0.54	0.0182	-0.16	1.0000				
ENSMUSG00000068082.12	*Grxcr1*	-2.76	0.0288	-1.52	1.0000				
ENSMUSG00000004892.13	*Bcan*	-1.33	0.0288	-0.43	1.0000				
ENSMUSG00000013584.5	*Aldh1a2*	-4.30	0.0304	-0.27	1.0000				
ENSMUSG00000036617.16	*Etl4*	-0.52	0.0306	-0.50	0.2724				
ENSMUSG00000100832.1	Gm29260	-0.60	0.0339	-0.63	0.0015				
ENSMUSG00000084901.6	Gm13266	-4.04	0.0375	-1.60	1.0000				
ENSMUSG00000016028.9	*Celsr1*	-0.78	0.0396	-0.53	0.8632				
ENSMUSG00000033726.8	*Emx1*	-0.76	0.0417	-0.23	1.0000				
ENSMUSG00000027967.8	*Neurog2*	-1.00	0.0432	-0.63	0.6869				
ENSMUSG00000023411.11	*Nfatc4*	-1.50	0.0438	-0.39	1.0000				
ENSMUSG00000033083.16	*Tbc1d4*	-0.65	0.0478	-0.49	0.8547				
