# Supplementary material for: Robust elimination of genome-damaged cells safeguards against brain somatic aneuploidy following Knl1 deletion
Source: Nat Commun. 2019 Jun 13;10:2588. doi: 10.1038/s41467-019-10411-w (PMC6565622; doi:10.1038/s41467-019-10411-w)
Supplement: Supplementary file 3 — Reporting Summary [file 41467_2019_10411_MOESM3_ESM.pdf]

nature research

Corresponding author(s): Kenneth Kwan

Last updated by author(s): Apr 28, 2019

Reporting Summary

Nature Research wishes to improve the reproducibility of the work that we publish. This form provides structure for consistency and transparency in reporting. For further information on Nature Research policies, see Authors & Referees and the Editorial Policy Checklist.

Statistics

For all statistical analyses, confirm that the following items are present in the figure legend, table legend, main text, or Methods section.

n/a	Confirmed	
☐	☒	The exact sample size (*n*) for each experimental group/condition, given as a discrete number and unit of measurement
☐	☒	A statement on whether measurements were taken from distinct samples or whether the same sample was measured repeatedly
☐	☒	The statistical test(s) used AND whether they are one- or two-sided<br>*Only common tests should be described solely by name; describe more complex techniques in the Methods section.*
☐	☒	A description of all covariates tested
☐	☒	A description of any assumptions or corrections, such as tests of normality and adjustment for multiple comparisons
☐	☒	A full description of the statistical parameters including central tendency (e.g. means) or other basic estimates (e.g. regression coefficient) AND variation (e.g. standard deviation) or associated estimates of uncertainty (e.g. confidence intervals)
☐	☒	For null hypothesis testing, the test statistic (e.g. *F*, *t*, *r*) with confidence intervals, effect sizes, degrees of freedom and *P* value noted<br>*Give P values as exact values whenever suitable.*
☒	☐	For Bayesian analysis, information on the choice of priors and Markov chain Monte Carlo settings
☒	☐	For hierarchical and complex designs, identification of the appropriate level for tests and full reporting of outcomes
☒	☐	Estimates of effect sizes (e.g. Cohen's *d*, Pearson's *r*), indicating how they were calculated

Our web collection on statistics for biologists contains articles on many of the points above.

Software and code

Policy information about availability of computer code

Data collection	*Provide a description of all commercial, open source and custom code used to collect the data in this study, specifying the version used OR state that no software was used.*
Data analysis	RNA-seq libraries were generated using a previously described ClickSeq procedure (Routh et al., 2015). RNA-seq data were subject to quality control check using FastQC v0.11.5 (https://www.bioinformatics.babraham.ac.uk/projects/download.html#fastqc). Adapter trimming was performed using cutadapt version 1.13 (http://cutadapt.readthedocs.io/en/stable/guide.html). Processed reads were aligned to the GENCODE GRCm38/mm10 reference genome (https://www.gencodegenes.org/mouse_releases/current.html) with STAR (v2.5.2a) and deduplicated according to UMI using UMI-tools (v0.5.3) (Dobin et al., 2013; Smith et al., 2017). Read counts were obtained with htseq-count (v0.6.1p1) with intersection-nonempty mode (Anders et al., 2015). Differential expression was determined with edgeR (Robinson et al., 2010). The p-value was calculated with likelihood ratio tests and the adjusted p-value for multiple testing was calculated using the Benjamini-Hochberg procedure, which controls false discovery rate (FDR). All RNA-seq data have been deposited in the GEO database (GSE114956).

For manuscripts utilizing custom algorithms or software that are central to the research but not yet described in published literature, software must be made available to editors/reviewers. We strongly encourage code deposition in a community repository (e.g. GitHub). See the Nature Research guidelines for submitting code & software for further information.

Data

Policy information about availability of data

All manuscripts must include a data availability statement. This statement should provide the following information, where applicable:

- Accession codes, unique identifiers, or web links for publicly available datasets
- A list of figures that have associated raw data
- A description of any restrictions on data availability

Provide your data availability statement here.

Field-specific reporting

Please select the one below that is the best fit for your research. If you are not sure, read the appropriate sections before making your selection.

☒ Life sciences ☐ Behavioural & social sciences ☐ Ecological, evolutionary & environmental sciences

For a reference copy of the document with all sections, see nature.com/documents/nr-reporting-summary-flat.pdf

Life sciences study design

All studies must disclose on these points even when the disclosure is negative.

Sample size

No statistical methods were used to predetermine sample size. Sample size was determined based on literature in the field and at least three animals per group for each analysis.

Data exclusions

For RNA-seq data, low quality reads and non-uniquely mapped reads were discarded. No data were excluded from any other analysis.

Replication

The reproducibility of our conclusions was verified by replication and orthogonal methods. RNA-seq data were validated using ddPCR and immunostaining. Pyknosis and CC3 were used to assess apoptosis. pKAP1 and gH2AX were used to assess DNA damage. Cortical area and thickness were used to assess brain size. ADGRE1 and AIF1 were used to characterize microglia.

Randomization

Randomization was not relevant to this study. Experimental groups were based on genotype.

Blinding

Blinding was not used for this study. The mutant phenotypes were robust and obvious at both macroscopic and microscopic levels of analysis. True blinding was not possible.

Reporting for specific materials, systems and methods

We require information from authors about some types of materials, experimental systems and methods used in many studies. Here, indicate whether each material, system or method listed is relevant to your study. If you are not sure if a list item applies to your research, read the appropriate section before selecting a response.

Materials & experimental systems

n/a	Involved in the study
☐	☒ Antibodies
☒	☐ Eukaryotic cell lines
☒	☐ Palaeontology
☐	☒ Animals and other organisms
☒	☐ Human research participants
☒	☐ Clinical data

Methods

n/a	Involved in the study
☒	☐ ChIP-seq
☒	☐ Flow cytometry
☒	☐ MRI-based neuroimaging

Antibodies

Antibodies used

The primary antibodies used in this study: rabbit anti-SOX2 (Millipore,1:500), rat anti-SOX2 (Thermo Fisher Scientific, 1:500), rat anti-EOMES (TBR2) (Thermo Fisher Scientific, 1:500), chicken anti-RBFOX3 (NEUN) (Millipore, 1:500), rabbit anti-cleaved Caspase-3 (CC3) (Cell Signaling Technology, 1:500), mouse anti-VIM (phospho-Ser55) (Enzo Life Sciences, 1:500), rat anti-BCL11B (Abcam, 1:500), rabbit anti-CUX1 (Santa Cruz Biotechnology, 1:250), rabbit anti-KI67 (Abcam,1:250), rabbit anti-p53 (Leica Microsystems, 1:250), rabbit anti-histone H2A.X (phospho-Ser139) (Cell Signaling Technology, 1:250), rabbit anti-IBA1 (AIF1) (Wako, 1:500), rabbit anti-ADGRE1 (F4/80) (Abcam, 1:500), goat anti-POU3F2 (BRN2) (Santa Cruz Biotechnology, 1:250), rabbit anti-TBR1 (Abcam, 1:500), rabbit anti-phospho KAP1(S824) (Bethyl, 1:500), mouse anti-SATB2 (Abcam, 1:500), rat anti-CldU (Accurate Chemical and Scientific Corporation, 1:250), chicken anti-GFP (Abcam,1:500), rabbit anti-GFP (Thermo Fisher Scientific, 1:500).

Validation

All antibodies that were used have been validated in previous published studies. These studies are listed in the manufacturers' websites.

Animals and other organisms

Policy information about studies involving animals; ARRIVE guidelines recommended for reporting animal research

Laboratory animals

Laboratory mice were used in this study. Mice of mixed genetic background were used. To generate the Casc5 conditional allele, a conditional-ready lacZ reporter Casc5 allele (Knl1tm1a(EUCOMM)Hmgu; EUCOMM (HEPD0665_2_E05)) was obtained from EUCOMM and recovered from cryopreserved spermatozoa at the University of Michigan Transgenic Core. The Casc5fl/fl conditional allele was generated by crossing with a Flpo recombinase transgene to remove the neomycin resistance gene

cassette from the germline via FRT site recombination. The Casc5fl/fl mice were used to generate conditional mutants. Additional mice used in this study are: p53 LoxP (The Jackson Laboratory, Stock No: 008462), ROSA26Flpo (The Jackson Laboratory, Stock No: 012930), hGFAP-Cre (The Jackson Laboratory, Stock No: 004600), Emx1-Cre (The Jackson Laboratory, Stock No: 005628). Animals of both sexes were used. The ages of the animals were as described. All experiments were carried out in accordance with a protocol approved by the University of Michigan Institutional Animal Care & Use Committee.

Wild animals	Wild animals were not used in this study.
Field-collected samples	Field-collected samples were not used in this study.
Ethics oversight	All experiments were carried out in accordance with a protocol approved by the University of Michigan Institutional Animal Care & Use Committee.

Note that full information on the approval of the study protocol must also be provided in the manuscript.
