# Supplementary material for: Robust elimination of genome-damaged cells safeguards against brain somatic aneuploidy following Knl1 deletion
Source: Nat Commun. 2019 Jun 13;10:2588. doi: 10.1038/s41467-019-10411-w (PMC6565622; doi:10.1038/s41467-019-10411-w)
Supplement: Supplementary file 2 — Peer Review [file 41467_2019_10411_MOESM2_ESM.pdf]

Editorial Note: This manuscript has been previously reviewed at another journal that is not operating a transparent peer review scheme. This document only contains reviewer comments and rebuttal letters for versions considered at *Nature Communications.*

REVIEWERS' COMMENTS:

Reviewer #1 (Remarks to the Author):

The authors have revised the manuscript thoroughly and satisfactory.

Reviewer #2 (Remarks to the Author):

In this revised manuscript for Nature Communications, the authors have substantially improved the manuscript with many new experiments as well as more fleshed out discussion. Together, the clarity of CASC5 mechanisms are improved and the significance of findings increased. While P53 is heavily studied in microcephaly, the authors make some interesting insights towards understanding its activation and downstream impact. The discovery that there are long term survival defects associated with aneuploidy independent of p53 is interesting. I just have a few remaining concerns.

1) In the initial review, concerns were raised about novelty in terms of p53 mechanisms and Casc5 biology. In response to prior suggestions, the authors reference a suggested paper regarding induced NPCs with a human CASC5 mutation. Although they reference the M phase data, it would also be important to note the common increase of aneuploidy and mitosis defects in NPCs. Further it would be valuable to discuss this paper to understand potential links with human and mouse NPCs.

2) Supplementary Figure 1F whole mount does not show sufficient resolution to conclude VZ expression of Casc5, as it only depicts expression in a thick part of the forebrain. This should be adjusted in the figure legend and in the main text.

3) Typo p. 17 reference to Fig. 8B should be 8c.

4) Authors claim phenotypic partial rescue differences showing t-tests of $p>0.05$ (ie. Fig 5a, b, 7e). However, when comparing 3 different genotypes, as they do here, this statistical test is not appropriate. They should use ANOVA analysis to compare all 3 genotypes first, followed by post hoc t test between individual genotypes to test for differences between these genotypes (and hence extent of rescue).

5) The rationale of using transcriptome to only evaluate surviving cells is over-simplified. The transcriptome inherantly compares at a population level so the mutant will have a different starting population (as their data suggest apoptosis is primarily limited to NPCs). I think this point should be included as a caveat of analysis. For example, if they had examined transcriptomes at E13.5, one may expect potentially more widespread changes, including p53 as well as cell fate markers.

Reviewer #3 (Remarks to the Author):

The authors provided a number of new experiments that have strengthened the study. It remains however the issue of novelty that in this case is quite low. Moreover, despite the new data, it is still unclear whether NPCs are the only or at lest main cell types that are affected in this mouse model.

Response to reviews

REVIEWERS' COMMENTS:

Reviewer #1 (Remarks to the Author):

The authors have revised the manuscript thoroughly and satisfactory.

> We thank this reviewer for important suggestions from the previous round of review.

Reviewer #2 (Remarks to the Author):

In this revised manuscript for Nature Communications, the authors have substantially improved the manuscript with many new experiments as well as more fleshed out discussion. Together, the clarity of CASC5 mechanisms are improved and the significance of findings increased. While P53 is heavily studied in microcephaly, the authors make some interesting insights towards understanding its activation and downstream impact. The discovery that there are long term survival defects associated with aneuploidy independent of p53 is interesting. I just have a few remaining concerns.

1) In the initial review, concerns were raised about novelty in terms of p53 mechanisms and Casc5 biology. In response to prior suggestions, the authors reference a suggested paper regarding induced NPCs with a human CASC5 mutation. Although they reference the M phase data, it would also be important to note the common increase of aneuploidy and mitosis defects in NPCs. Further it would be valuable to discuss this paper to understand potential links with human and mouse NPCs.

> We thank this reviewer for the suggestion. We now reference this work in both Results and Discussion to highlight similarities in mitosis defects and cell death in human and mouse NPCs in the absence of CASC5 function.

2) Supplementary Figure 1F whole mount does not show sufficient resolution to conclude VZ expression of Casc5, as it only depicts expression in a thick part of the forebrain. This should be adjusted in the figure legend and in the main text.

> We agree with this and have made the appropriate changes in the figure legend and the text.

3) Typo p. 17 reference to Fig. 8B should be 8c.

> We have made this correction.

4) Authors claim phenotypic partial rescue differences showing t-tests of p>0.05 (ie. Fig 5a, b, 7e). However, when comparing 3 different genotypes, as they do here, this statistical test is not appropriate. They should use ANOVA analysis to compare all 3 genotypes first, followed by post hoc t test between individual genotypes to test for differences between these genotypes (and hence extent of rescue).

> We agree. We have now used the suggested ANOVA with post-hoc *t* test for all comparisons of three genotypes and updated Figures 5, 6, 7 and Supplementary Figures 5, 6 to reflect these changes.

5) The rationale of using transcriptome to only evaluate surviving cells is over-simplified. The transcriptome inherantly compares at a population level so the mutant will have a different starting

population (as their data suggest apoptosis is primarily limited to NPCs). I think this point should be included as a caveat of analysis. For example, if they had examined transcriptomes at E13.5, one may expect potentially more widespread changes, including p53 as well as cell fate markers.

> We have more clearly described population changes in the transcriptome following *Casc5* deletion. We noted a decrease in cell type markers for the excitatory neuron lineage, and an increase in cell type markers for the inhibitory neuron lineage. These data are consistent with loss of NPCs from the *hGFAP-Cre* lineage to apoptosis following *Casc5* deletion, and a relative increase in the inhibitory neuron lineage from which *Casc5* was not deleted, thus reflecting expected population level changes in the cell type composition of the cKO brain. We do not, however, have data on whether E13.5 transcriptome changes would be more widespread in comparison.

Reviewer #3 (Remarks to the Author):

The authors provided a number of new experiments that have strengthened the study. It remains however the issue of novelty that in this case is quite low. Moreover, despite the new data, it is still unclear whether NPCs are the only or at lest main cell types that are affected in this mouse model.

> We are happy to clarify. *Casc5* is exclusively expressed in dividing cells and encodes a protein that functions in the SAC, a mitotic checkpoint. In *Casc5* cKO, we found chromosome missegregation and DNA damage in mitotic NPCs, and widespread p53 activation in SOX2+ NPCs. Together, previous work (including the human NPC work suggested by this reviewer) and our results convergently support NPC mitosis as the point of primary deficit following *Casc5* deletion. It is possible, however, for apoptosis to occur post-mitosis after initial mitotic errors. We have included this point in the Discussion.
